# Iatrogenic air embolism: pathoanatomy, thromboinflammation, endotheliopathy, and therapies

**DOI:** 10.3389/fimmu.2023.1230049

**Published:** 2023-09-19

**Authors:** Phillip L. Marsh, Ernest E. Moore, Hunter B. Moore, Connor M. Bunch, Michael Aboukhaled, Shaun M. Condon, Mahmoud D. Al-Fadhl, Samuel J. Thomas, John R. Larson, Charles W. Bower, Craig B. Miller, Michelle L. Pearson, Christopher L. Twilling, David W. Reser, George S. Kim, Brittany M. Troyer, Doyle Yeager, Scott G. Thomas, Daniel P. Srikureja, Shivani S. Patel, Sofía L. Añón, Anthony V. Thomas, Joseph B. Miller, David E. Van Ryn, Saagar V. Pamulapati, Devin Zimmerman, Byars Wells, Peter L. Martin, Christopher W. Seder, John G. Aversa, Ryan B. Greene, Robert J. March, Hau C. Kwaan, Daniel H. Fulkerson, Stefani A. Vande Lune, Tom E. Mollnes, Erik W. Nielsen, Benjamin S. Storm, Mark M. Walsh

**Affiliations:** ^1^Department of Emergency Medicine, Saint Joseph Regional Medical Center, Mishawaka, IN, United States; ^2^Department of Surgery, Ernest E. Moore Shock Trauma Center at Denver Health and University of Colorado Health Sciences Center, Denver, CO, United States; ^3^University of Colorado Health Transplant Surgery - Anschutz Medical Campus, Aurora, CO, United States; ^4^Department of Emergency Medicine, Henry Ford Hospital, Detroit, MI, United States; ^5^Indiana University School of Medicine, South Bend, IN, United States; ^6^Department of Emergency Medicine, Goshen Health, Goshen, IN, United States; ^7^Department of Family Medicine, Saint Joseph Health System, Mishawaka, IN, United States; ^8^Department of Trauma & Surgical Research Services, South Bend, IN, United States; ^9^Department of Emergency Medicine, Beacon Health System, Elkhart, IN, United States; ^10^Department of Internal Medicine, Mercy Health Internal Medicine Residency Program, Rockford, IL, United States; ^11^Department of Cardiovascular and Thoracic Surgery, RUSH Medical College, Chicago, IL, United States; ^12^Division of Hematology and Oncology, Department of Medicine, Northwestern University, Chicago, IL, United States; ^13^Department of Emergency Medicine, Naval Medical Center Portsmouth, Portsmouth, VA, United States; ^14^Research Laboratory, Nordland Hospital, Bodø, Norway; ^15^Faculty of Medicine, Institute of Clinical Medicine, University of Oslo, Oslo, Norway; ^16^Department of Immunology, Oslo University Hospital, University of Oslo, Oslo, Norway; ^17^Department of Anesthesia and Intensive Care Medicine, Surgical Clinic, Nordland Hospital, Bodø, Norway; ^18^Institute of Clinical Medicine, University of Tromsø, Tromsø, Norway; ^19^Faculty of Nursing and Health Sciences, Nord University, Bodø, Norway

**Keywords:** air embolism, decompression sickness, hyperbaric oxygenation, thromboinflammation, microbubbles, arterioles

## Abstract

Iatrogenic vascular air embolism is a relatively infrequent event but is associated with significant morbidity and mortality. These emboli can arise in many clinical settings such as neurosurgery, cardiac surgery, and liver transplantation, but more recently, endoscopy, hemodialysis, thoracentesis, tissue biopsy, angiography, and central and peripheral venous access and removal have overtaken surgery and trauma as significant causes of vascular air embolism. The true incidence may be greater since many of these air emboli are asymptomatic and frequently go undiagnosed or unreported. Due to the rarity of vascular air embolism and because of the many manifestations, diagnoses can be difficult and require immediate therapeutic intervention. An iatrogenic air embolism can result in both venous and arterial emboli whose anatomic locations dictate the clinical course. Most clinically significant iatrogenic air emboli are caused by arterial obstruction of small vessels because the pulmonary gas exchange filters the more frequent, smaller volume bubbles that gain access to the venous circulation. However, there is a subset of patients with venous air emboli caused by larger volumes of air who present with more protean manifestations. There have been significant gains in the understanding of the interactions of fluid dynamics, hemostasis, and inflammation caused by air emboli due to *in vitro* and *in vivo* studies on flow dynamics of bubbles in small vessels. Intensive research regarding the thromboinflammatory changes at the level of the endothelium has been described recently. The obstruction of vessels by air emboli causes immediate pathoanatomic and immunologic and thromboinflammatory responses at the level of the endothelium. In this review, we describe those immunologic and thromboinflammatory responses at the level of the endothelium as well as evaluate traditional and novel forms of therapy for this rare and often unrecognized clinical condition.

## 1 Introduction

The word “embolus” derives from a Greek word referring to a stopper (1). Therefore, a vascular air embolus is a bubble that stops the flow of blood in the arteries and/or veins. Iatrogenic vascular air embolism is a relatively infrequent event but can be associated with significant morbidity and mortality, and thus, early recognition and treatment are crucial. The clinical consequences of iatrogenic air embolism depend on the anatomic locations and whether the emboli are venous or arterial. Most clinically significant iatrogenic air emboli are caused by arterial obstruction of small vessels because the pulmonary filtration absorbs the more frequent, smaller volume bubbles that gain access to the venous circulation. However, there is a subset of patients with venous air emboli caused by larger bubbles who present with more protean manifestations. The knowledge of iatrogenic air emboli has expanded significantly since the late 19th century, yet early recognition of this disease remains underappreciated (2–7).

Arterial and venous air emboli include those caused by non- iatrogenic phenomena, the most clinically frequent being changes in ambient pressure during diving resulting in decompression illness (DCI), whereby dissolved gasses, primarily nitrogen, come out of solution and form bubbles within the body (8). Therefore, the term “gas” is often used when referring to diving-related gas emboli. However, in iatrogenic cases, most gas emboli are due to the introduction of ambient air into the vascular circulation. This review will use the terms venous air embolism (VAE) and arterial air embolism (AAE) to specify air in the venous and arterial circulations, respectively, as opposed to the more general term “gas.” It has been shown that the degree of complement activation is independent of the type of gasses present because air, pure oxygen, and pure nitrogen activate complement to the same extent (1–3, 9–16).

Historically, reports of air embolism have existed since 1769 when the Italian anatomist Giovanni Morgagni discussed cerebral air embolism (CAE) in his postmortem findings (17). However, animal experiments have been performed since 1667 when Francesco Redi injected air intravenously into different animals, which subsequently killed them (5). Experimental studies performed during the 250 years after Morgagni’s findings have provided valuable data concerning the manifestations of VAE and AAE. In the late 19th and early 20th centuries, increased reporting of air emboli occurred with the observation that the supine and head-down position improved outcome (1–3, 5, 6, 12, 18). In 1947, Thomas Durant proposed Durant’s maneuver, which involved Trendelenburg and left lateral decubitus (LLD) positioning of the patient as a therapeutic maneuver for suspected VAE to prevent right ventricular outflow obstruction. Durant also demonstrated that CAE could cause local brain ischemia (15). Other than Durant’s maneuver and the administration of 100% oxygen *via* non-rebreather, there was minimal effective therapy available until the 1970s when the use of hyperbaric oxygen therapy (HBOT) was introduced as the preferred treatment for recompression of air emboli (1, 19–21). In the following two decades, the pace of research increased significantly because of the increased availability of ultrasound and new tools for diagnosing the immunologic and hemostatic consequences of vascular air emboli (1, 21–24).

Single cases and multi-institution case series have described the complications of air embolism that can arise in a multitude of clinical scenarios including radiologic interventions, polytrauma, and surgical cases. Specifically, air emboli have been described in neurosurgery, abdominal surgery, genitourinary instrumentation, vascular surgery, cardiac surgery, and liver transplant surgery. Endoscopy, hemodialysis, thoracentesis, tissue biopsy, angiography, and central and peripheral venous access and removal have joined surgery and trauma as significant causes of VAE (2, 4, 25). With the expansion of interventional radiology (IR), air emboli have become much more common in these types of procedures. Air embolism has been previously reported in the IR literature at a frequency of about 0.13% (4, 26). They have an estimated incidence of 1 in 772 endovascular procedures according to one series. It has also been proposed that iatrogenic air embolism complicates 2.65 in 100,000 hospitalizations. The true incidence may be greater since many air emboli are asymptomatic (14, 26–28).

There have been advances in the understanding of the interactions of fluid dynamics, hemostasis, and inflammation caused by air emboli due to *in vitro* and *in vivo* studies of bubbles in small vessels, including recent research regarding the thromboinflammatory changes at the level of the endothelium (12, 29–50).

Basic treatment of air embolism will involve HBOT (51). However, other treatment measures in conjunction with HBOT are useful and can lead to better patient outcomes. Although air emboli due to invasive non-surgical procedures are considered uncommon, the severe symptoms and relative preventability make elucidating both treatment and prevention of air emboli useful (2–4, 27, 52, 53). Several experimental animal studies have shed light on the prevention, treatment, and potential clinical course of air embolisms (3, 54–57). It has been previously described that air in the cerebral arterial circulation may cause an immediate increase in intracranial pressure (ICP) resulting in an “extremely inhomogeneous distribution” of cerebral blood flow (CBF) with localized heterogeneous ischemia and hyperemia (3). This is thought to be the cause of the recurrence of symptoms with resumption of ambient pressure after HBOT, which is well-described in the literature (50, 58, 59). Here, we review the anatomical and pathophysiologic foundations responsible for the varied clinical presentations, diagnoses, and treatments of iatrogenic air embolisms.

## 2 Pathoanatomy and importance of patient positioning

**Figure 1:**
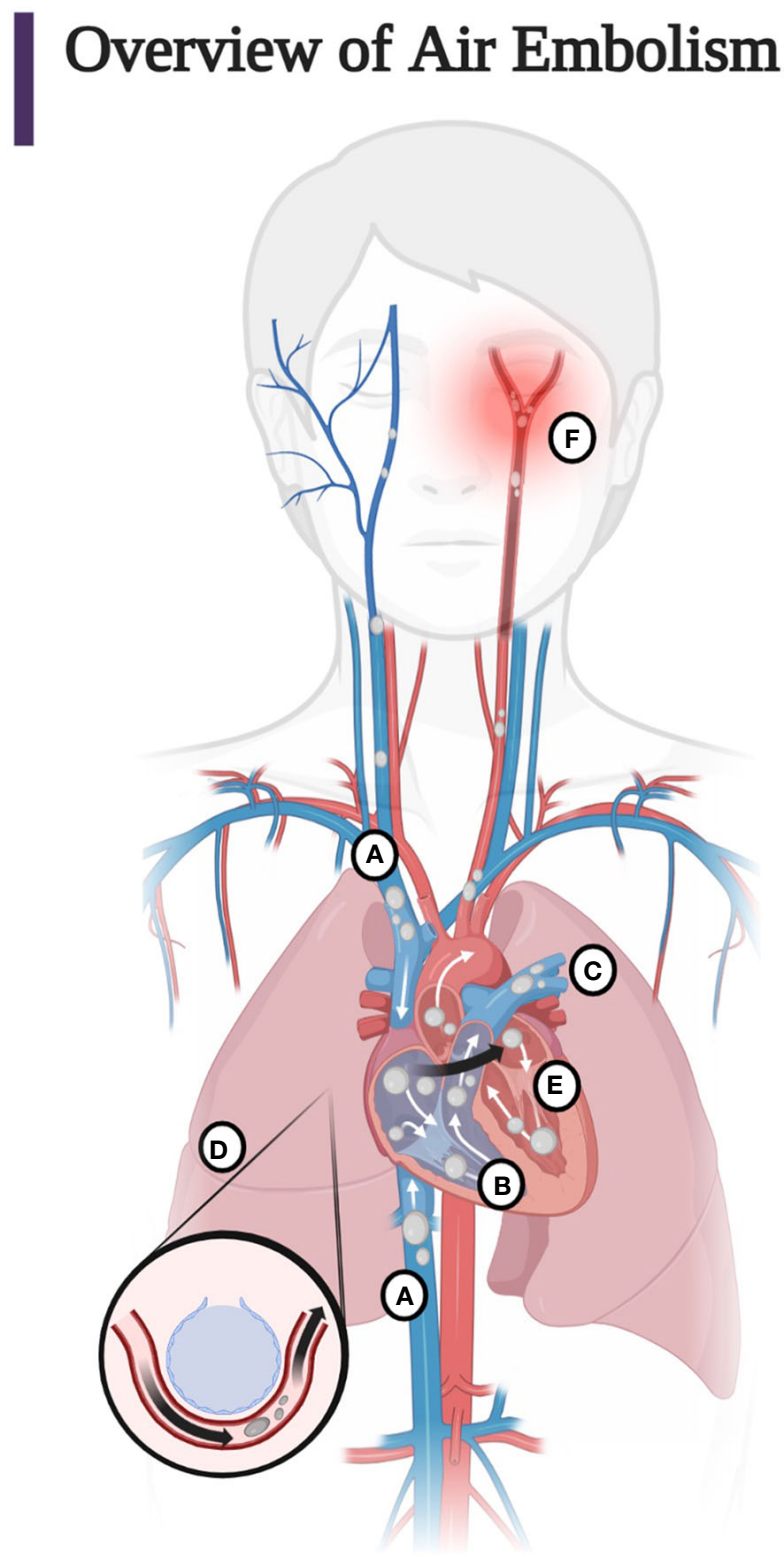
**(A)** Entry of air into the superior and inferior vena cava. Most air follows the superior or inferior vena cava into the right ventricle. Less commonly, air injected into the venous circulation will rise in a retrograde fashion into the cerebral circulation due to the natural buoyancy of the air bubble and cause protean symptoms of venous occlusion of the cerebral vasculature. This scenario occurs, for example, with the injection of air during the insertion or withdrawal of a central line in the sitting position. Note that due to the competing effects of buoyancy and drag, larger bubbles are more likely than smaller bubbles to become retrograde (60, 61). **(B)** Air in the right ventricle may totally occlude the pulmonary outflow tract causing an “air lock” or “vapor lock” with resultant shock. **(C)** Air may pass through the pulmonary artery, may diffuse into the alveoli, or be trapped in the pulmonary filter causing inflammation with impairment of gas exchange. **(D)** Air may overwhelm the pulmonary circulation and enter the left heart and systemic circulation or traverse a patent foramen ovale or a right-to-left shunt (arrow). **(E)** Air may enter the left heart and systemic circulation directly by injection into the pulmonary vein or from the right heart *via* the lung, a right-to-left shunt, or a patent foramen ovale. **(F)** Systemic air embolism, whether from the right heart or injected directly into the arterial circulation causes end organ damage, most commonly cerebral and cardiac. (Adapted from Storm 2022) (Created with BioRender.com).

Understanding the pathophysiology of air embolisms is best approached by following the path of air injected into the venous or arterial circulation. The clinical manifestations of the introduction of air into the vasculature are a function of the location, volume, and rate of injection of air, which determine the pathway taken by the air (**Figure 1**) (1–4, 13, 14, 62, 63).

Animal studies have demonstrated that the volume and rate of air injected into the venous circulation correlate with signs and symptoms. Bubbles can cross the pulmonary capillary bed and enter arterial circulation if they overwhelm pulmonary filtration capacity (64). In canine experiments, bubbles that were too small to get trapped (smaller than 22 µm) could enter directly into arterial circulations and provoke inflammation as they passed through the pulmonary circulation (65). In the same animal experiments, it was shown that at infusion rates of a single bolus of over 20 cc of gas or 0.15 cc/kg/min for 30 minutes, the bubbles may deform or rupture the lung capillaries, inflame the pulmonary endothelium, and cross into the pulmonary vein and into the systemic arterial circulation (64, 66). Once the bubble arrives on the arterial side of the circulation, the bubbles can lodge in an end organ, most lethally in the cerebral or coronary circulation. Lower volumes of air injected into the human venous circulation (less than 50 cc) may be absorbed by the pulmonary filtration system without pathologic response (2). Larger amounts of air may also be absorbed by the pulmonary filtration system but may also become trapped in the pulmonary circulation causing pulmonary edema and inflammation but are more likely to exceed pulmonary filtration capacity or pass through a right-to-left shunt resulting in paradoxical embolism. In any large volume symptomatic VAE, an “air lock” or “vapor lock” may be induced in the right ventricular outflow tract which may produce circulatory collapse. An AAE may further develop depending on the natural evolution of the “air lock” and response to treatment (2–4, 12–14, 62, 64). In case reports, accidental injection of 200–300 cc of air into the human vascular circulation has been described as lethal (4).

For clarity, we will describe the effects of VAE, paradoxical air embolism, and direct AAE separately.

### 2.1 Venous air embolism

**Figure 2:**
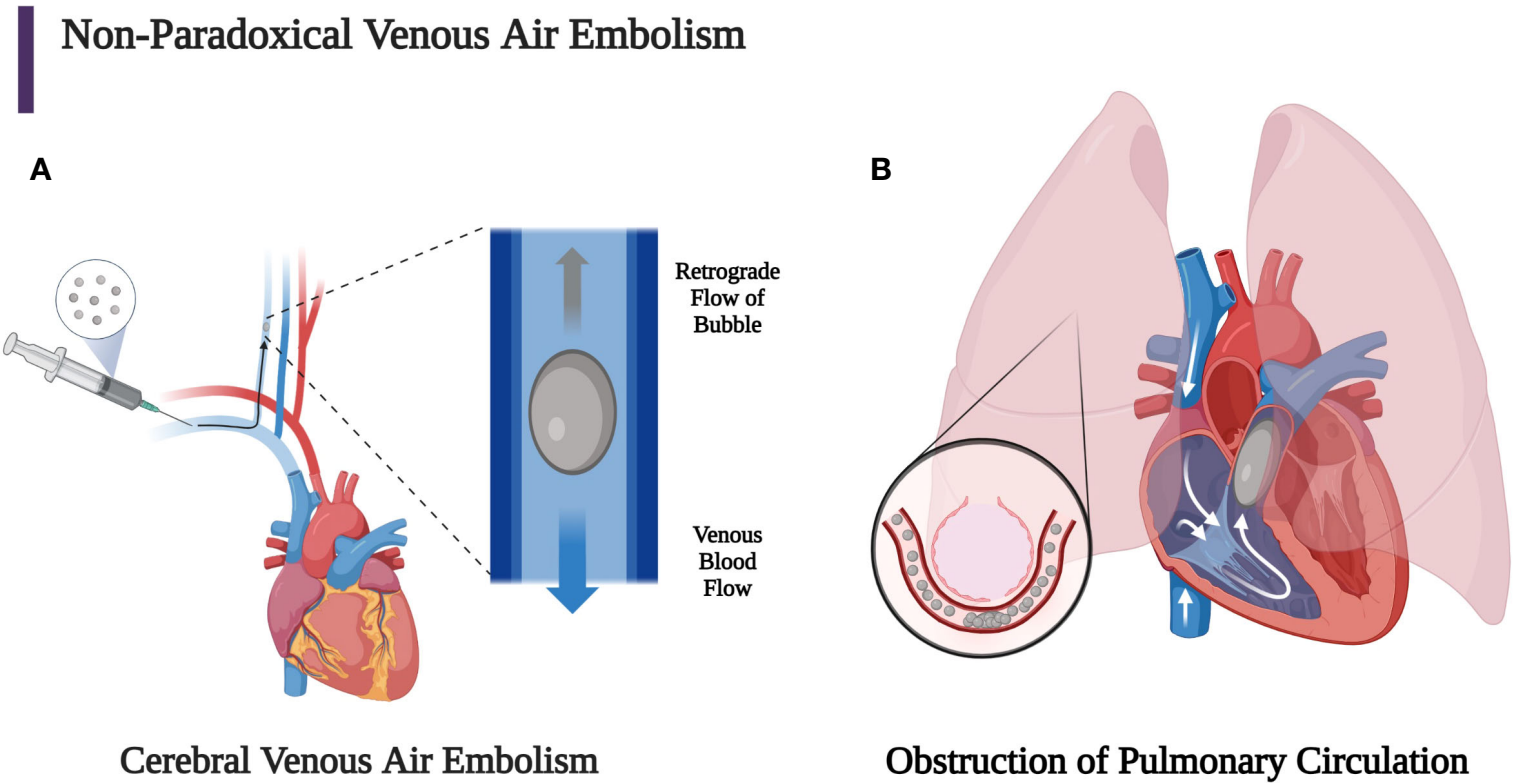
**(A)** Bubbles that stay in the venous circulation may proceed retrogradely towards the head if the patient remains in a sitting position since the buoyancy of the bubble can overcome slow antegrade venous blood flow. Larger bubbles are more buoyant than smaller bubbles, so they are more likely to experience retrograde flow, which can allow the bubble to reach the brain and cause a venous cerebral air embolism. This can lead to mental status change, seizure, focal neurological deficit, and shock. **(B)** Venous air emboli may also travel through the superior or inferior vena cava into the heart, which can lead to obstruction of the right ventricular outflow tract if the bubble is large enough. Smaller bubbles may obstruct pulmonary capillaries, leading to pulmonary vasoconstriction, obstruction, and capillary leak (2, 3, 29, 60, 67). (Created with BioRender.com).

VAE is caused by air being introduced directly into the venous circulation and may follow one or more of primarily three pathways. The VAE may ascend superiorly through the valveless superior vena cava and the jugular veins to cause CAE. The bubble can travel against blood flow in the veins and towards the head if its buoyancy allows its speed to exceed slow venous blood flow. This is known as retrograde flow (**Figure 2A**). Large bubbles are more buoyant, so they are more likely to travel to the brain if the patient is seated upright (60, 61). Durant’s maneuver, in concert with mechanical removal of air through aspiration performed within minutes of VAE identification, may mitigate these effects (4, 15, 62, 68–70). The most common causes of cerebral VAE are central lines or, less frequently, peripheral lines.

The other two primary routes by which VAE may travel are to the inferior vena cava to the liver (in ideal Durant’s positioning) or to the right heart and pulmonary circulation. Large volumes of bubbles (greater than 50 cc) may overwhelm the capacity of the lung to clear the gas and cause an “air lock” or “vapor lock” in the right ventricle. The resultant reduction of flow from the right heart may cause an increase of central venous pressure (CVP) and decreased pulmonary and systemic arterial pressure. A large volume of smaller bubbles can traverse the right ventricular outflow tract and lodge in the pulmonary arterioles or microcirculation reducing blood flow and causing vasoconstriction, further increasing right ventricular pressure and pulmonary vascular resistance. Whether small or large bubbles are injected into the venous circulation, a percentage of the bubbles may pass through the pulmonary capillaries and may enter the arterial circulation causing organ ischemia due to AAE (**Figure 2B**). The secondary immunothrombotic effects of air embolism can result in significant local endothelial damage. The accumulation of fibrin, platelets, and activated neutrophils occurs at the gas-fluid interface. Subsequent endothelial injury is then caused by complement activation and the release of reactive oxygen species, inflammatory cells, and mediators. This endothelial damage may cause non-cardiogenic pulmonary edema, bronchoconstriction, hypoxemia due to ventilation perfusion mismatching, and increased physiologic dead space with decreased lung compliance and increased airway resistance (2–4, 13, 14, 62, 71–77).

Animal studies have investigated how the pulmonary filtration system protects the systemic circulation, including cerebral and coronary circulations. These studies have defined the volume and rate of bubbles that can overwhelm this pulmonary filtration system and have provided insight into the thromboinflammatory changes at the level of the pulmonary endothelium, which produces the clinical response seen with varying volumes of air injected into the venous and arterial circulation (2–4, 50, 65).

Canine studies have shown that the pulmonary filtration threshold leading to arterial spillover of air bubbles in over 70% of dogs is 0.40 cc of air/kg/min (78). When VAEs are entrapped in the pulmonary circulation, they can lodge in pulmonary capillary beds compromising gas exchange. This may not only cause diminished gas exchange but also cardiac arrhythmias, dyspnea, pulmonary hypertension, and acute cor pulmonale. The entrapment of the pulmonary air bubbles may also result in pulmonary tissue injury with capillary leak. The resultant reduction of the functionality of endogenous surfactant also causes impaired gas exchange due to alveolar collapse and atelectasis, which often requires mechanical ventilation (3, 79). Endothelial and pulmonary cellular injury and lung edema are the result of thromboinflammatory changes provoked by the release of damage-associated molecular patterns, normally not exposed to the innate immune system but recognized by the pattern recognition receptors when cell and tissue damage occur. Innate upstream systems like complement, toll-like receptors, and others induce downstream release (e.g., from the complement C5a-C5aR1 axis) of innumerable of inflammatory vasoactive mediators such as thromboxane, leukotrienes, cytokines, and coagulation factors with resultant increase in alveolocapillary permeability (1, 3, 12, 30, 35–37, 79, 80).

Symptomatic VAEs with protean prodromes such as arrhythmias, chest pain, dyspnea, nausea, dizziness, blurry vision, syncope, and shock occur much less frequently than symptomatic AAEs that show similar manifestation yet are accompanied by symptoms and signs of focal, cerebral, and cardiac ischemia. Because air injected into the venous circulation can be trapped in the right heart, it has been suggested that the assumption of the Trendelenburg position in the LLD allows for dissipation of the bubbles outside of the pulmonary outflow tract. Therefore, Durant’s maneuver’s use is only for clinical situations where there is evidence of air trapping in the right heart, so it is reserved for VAE and is not to be used for AAE (10, 13, 60, 68, 81–83).

### 2.2 Arterial air embolism

#### 2.2.1 Paradoxical air embolism

**Figure 3:**
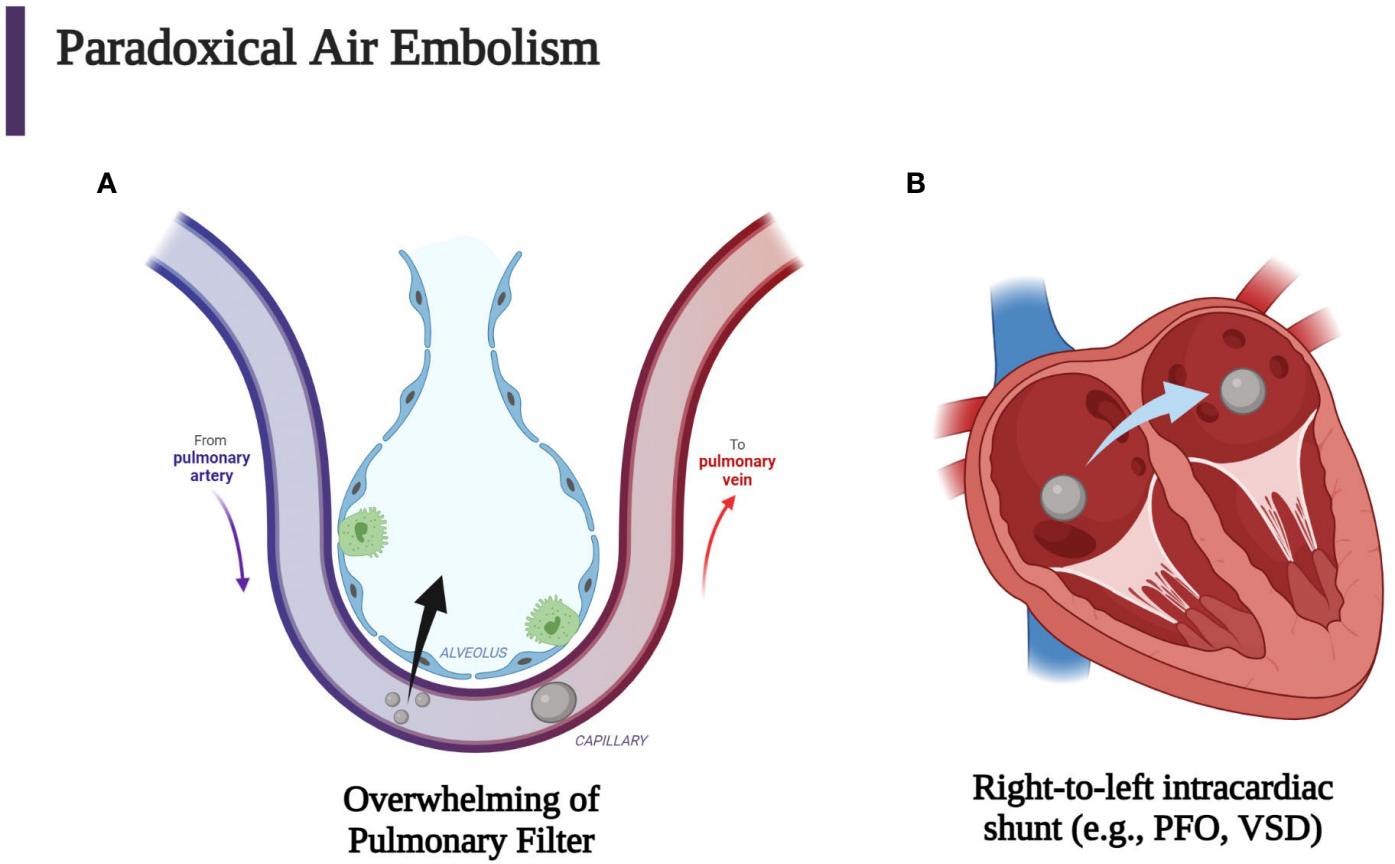
Iatrogenic paradoxical air embolism can occur when air enters the venous circulation with subsequent entrance into the systemic arterial circulation which then causes end-artery occlusion. **(A)** Venous air may overwhelm the pulmonary circulation either through a large bolus of gas or small continuous amounts. **(B)** Another mechanism for entrance of venous air into the arterial circulation is the passage of air through a patent foramen ovale (PFO), a ventricular septal defect (VSD), or another right-to-left cardiac or pulmonary shunt. Every venous air embolism has the potential to evolve into an arterial air embolism (2, 3, 84). (Created with BioRender.com).

Air in the venous circulation may overwhelm the pulmonary filter and enter the arterial circulation (**Figure 3A**) (84). These paradoxical emboli can also occur in the presence of any right-to- left shunt and can be enhanced by positive end-expiratory pressure and Valsalva maneuvers (**Figure 3B**). Thus, the pathway for air to travel once injected into the venous circulation depends on the pressure gradients, volume, and rate of air injected (2–4, 13, 14, 62, 64).

#### 2.2.2 Direct arterial air embolism

**Figure 4:**
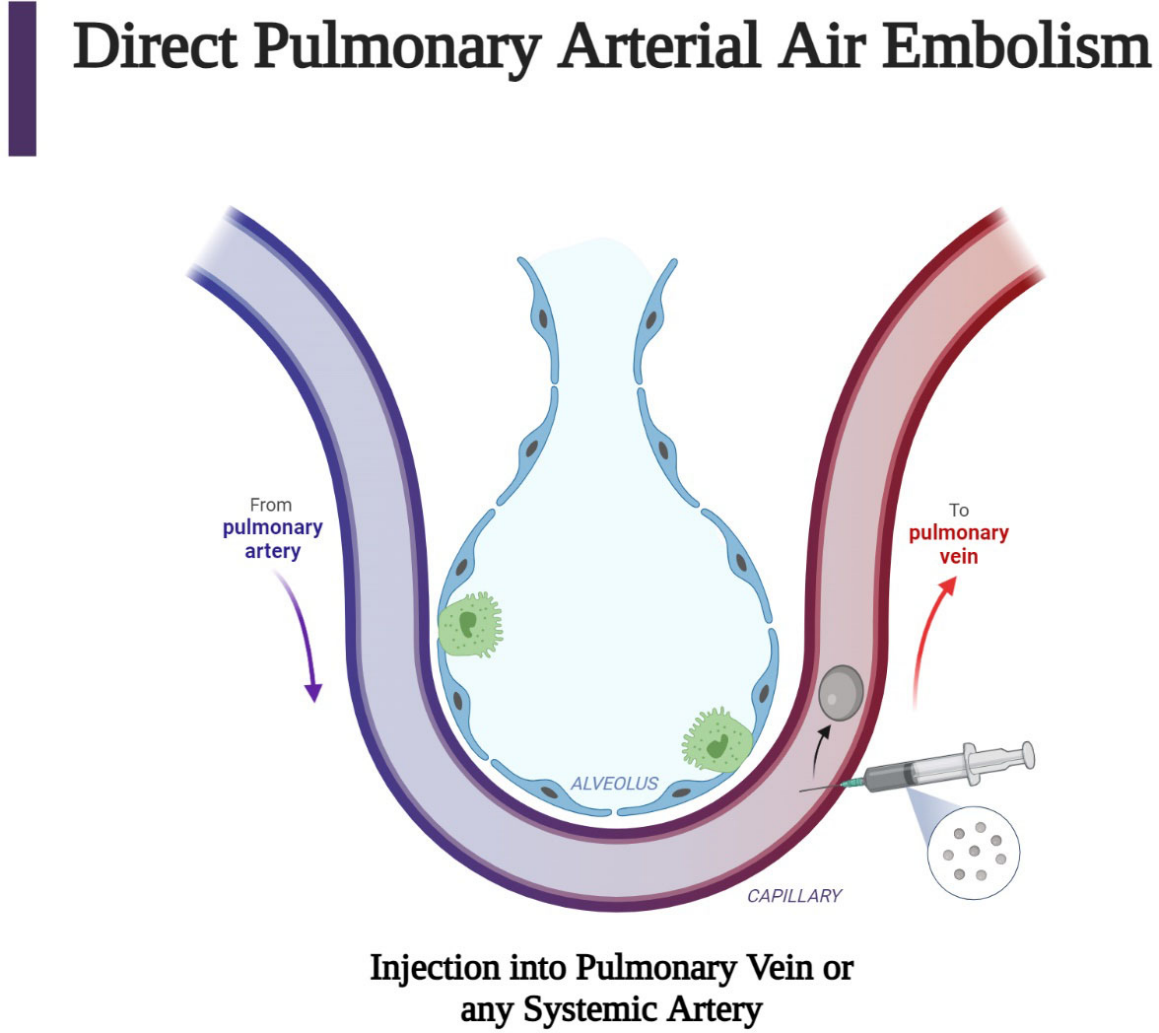
Direct air embolism is caused by the entry of gas into the pulmonary veins or directly into the arteries of the systemic circulation. This figure describes the injection of air into pulmonary venous circulation, which can occur after a procedure such as a biopsy. Subsequent arterial air embolization can occur immediately after the biopsy (63). (Created with BioRender.com).

Examples of direct AAE include inadvertent injection of air into the pulmonary veins or any systemic arterial side of circulation (**Figure 4**). Injection of air into the pulmonary veins, for example, may cause symptoms depending on the volume of air and the anatomic destination of the air bubble. Travel to the carotid and coronary arteries may cause immediate stroke or cardiac-related symptoms. Similar symptoms may occur due to direct injection during carotid surgery, during cardiac catheterization with inadvertent injection of air into the coronary arteries, or with perforation of the left atrium and creation of an atrial-esophageal fistula.

Historically, presentations of coronary air embolism were noted by Durant in 1949 (16), whereby within minutes of injection, a hypertensive crisis and ventricular fibrillation ensued. This canine study reported that doses between 0.05 and 1 cc of air injected into the left anterior descending coronary artery caused significant mortality (16). Durant’s initial findings in canines have been supported by subsequent studies on anesthetized swine wherein air bubbles with diameters of 150 µm and volumes of 0.002 cc/kg of body weight were injected into coronary circulation and caused severe life-threatening arrhythmias and signs of global cardiac dysfunction (3, 85, 86). It was also noted that mongrel dogs injected with 0.02 cc/kg of air into the left anterior descending coronary artery had a 28% mortality without extracorporeal circulation, whereas the animals who were on extracorporeal circulation had no post-embolic death. These findings were supported by the absence of myocardial ischemia in the extracorporeal circulation group as assessed by continuous thermographic measurements and fluorescent techniques (87).

Occlusion of arterial cerebral vessels is the more common manifestation of AAE because of the predominance of arterial flow, which bypasses the coronary vessels as the bubbles follow the higher flow into the cerebral circulation. Any procedure that allows for the entrance of air into the arterial circulation will result in nearly immediate symptoms due to cerebral arterial obstruction. As mentioned before, this obstruction causes an immediate increase in ICP resulting in a heterogeneous distribution of CBF with localized heterogeneous ischemia and hyperemia. Abnormal distribution of CBF can explain the significant vacillation of symptoms before, during, and after HBOT (3, 54–57).

### 2.3 Decompression illness and pulmonary barotrauma

DCI broadly describes two entities: decompression sickness (DCS) and direct pulmonary barotrauma causing arterial gas embolism (AGE). The term AGE is used rather than AAE in diving to include breathing gas mixtures which may include varying amounts of oxygen, nitrogen, and occasionally helium. DCS occurs when bubbles of gas form in tissues of the body following a reduction of environmental pressure causing vascular and tissue injury. It manifests the symptoms colloquially termed the “bends,” “chokes,” or “staggers” as a consequence of bubble formation from dissolved inert gas during or after decompression (88). The pathophysiological rationale for the treatment of iatrogenic vascular air emboli is primarily based on the better studied DCI in diving but also in aviation and space flight. Henry’s law (C = kP) states that the concentration of dissolved gas (C) is equal to the product of Henry’s constant (k) and the partial pressure of the gas (P) (89). This conceptualizes the dissolving and formation of bubbles in and out of solution with changes in the partial pressure of the gas. Because partial pressure of a gas is proportional to total absolute pressure, decreasing ambient pressure will drive gas out of solution and vice versa. These bubbles form within the venous circulation rather than arterial due to lower partial pressures and most often remain asymptomatic because of pulmonary capillary absorption. Much as with iatrogenic VAE, larger volumes of bubbles may cause symptoms through entrapment in the pulmonary circulation or arterial manifestations *via* paradoxical embolism. Additionally, rapid decreases in ambient pressure during ascent among divers may cause the entrance of gas into the pulmonary venous circulation, which causes an AGE. Pulmonary barotrauma due to expansion of trapped air in the alveoli with reference to Boyle’s Law (P1V1 = P2V2) results in alveolar rupture and translocation of air directly into the pulmonary venous circulation (63). This can happen, in particular, if an ascending diver holds their breath during ascent (12). A large body of evidence has accumulated in studies of DCI among divers; therefore, discussion of DCI forms a useful foundation for the pathophysiologic understanding of the clinical presentation caused by the rare event of the introduction of iatrogenic air bubbles into the venous or arterial circulations (12, 88, 90).

**Figure 5:**
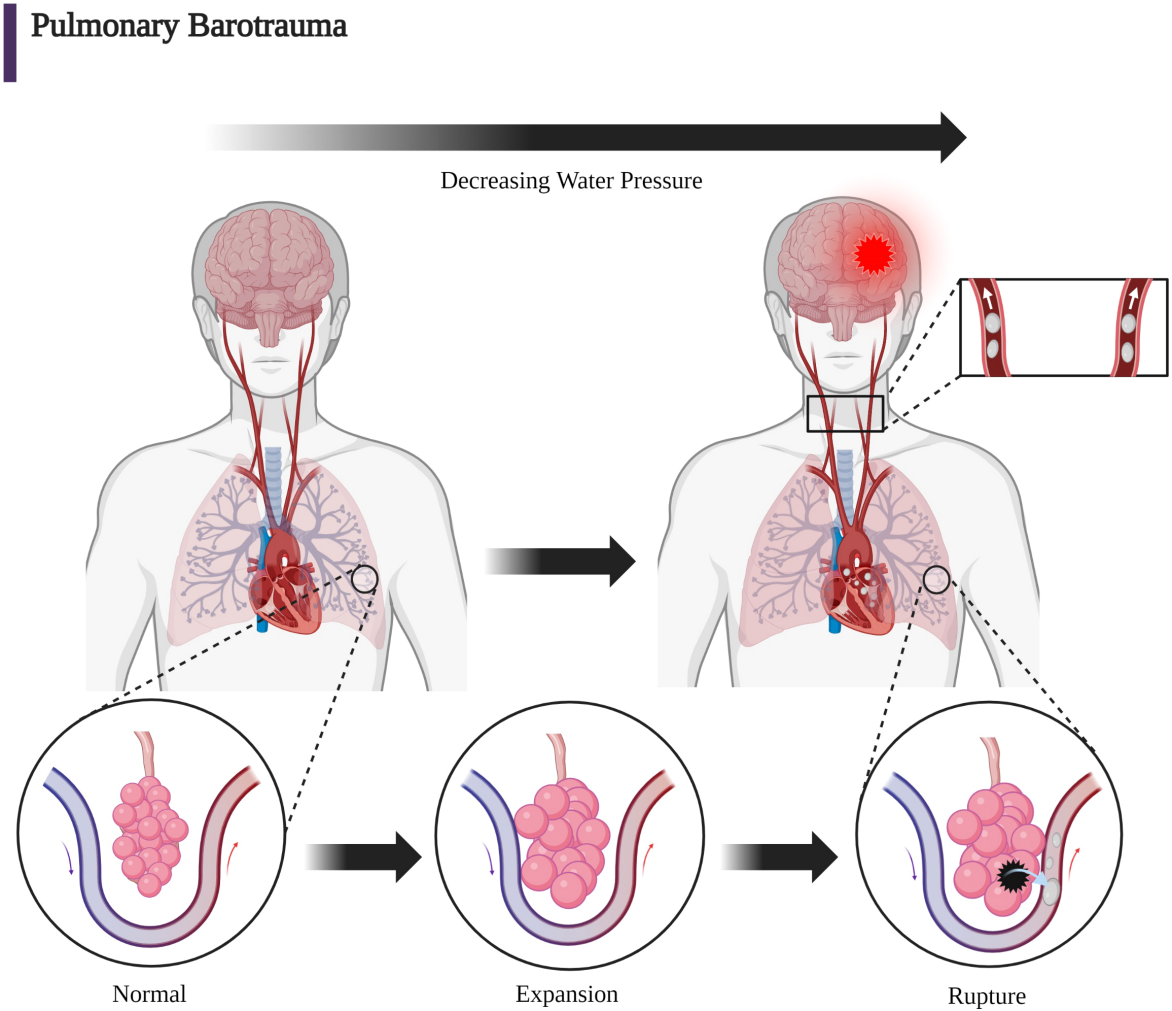
The mechanical forces due to increasing barometric pressure expressed on the alveolar wall can cause infiltration into the pulmonary venous circulation. With respect to DCI, Boyle’s law (P_1_V_1_ = P_2_V_2_) can give mathematical insight to the etiology of baro-rupture. As pressure decreases during ascent, volume of air in the lungs increases. This is why rapid ascension coupled with slow exhalation (or holding one’s breath) can lead to a disastrous scenario (63, 90). (Created with BioRender.com).

AGE may occur not only after decompression but also by iatrogenic or pulmonary physical barotrauma from blunt or penetrating injury. For example, alveolar or bronchiolar rupture with resultant introduction of air into the arterial circulation may occur with noninvasive positive pressure or mechanical ventilation following blunt or penetrating lung trauma. Much as with diving- associated entrance of air into the pulmonary venous circulation, the entrance of air into the systemic arterial circulation may cause evidence of end-organ arterial ischemia, which is most commonly cerebral injury (67, 91–95). Clinical descriptions of DCI were written as early as 1854 regarding the digging of coal pits (12, 96). Compressor technology required for diving developed in the 1880s and resulted in the description of the symptom complex known as the “bends” (12). The term “bends” derived from caisson workers who presented with pulmonary, neurologic, and vestibular anomalies while working on bridge projects in the 1870s. Classically, the clinical description of DCI has been a function of the major anatomic area which is affected by air bubbles. Clinical presentations of DCI are divided into neurologic, inner ear, and pulmonary findings. Specifically, pulmonary involvement such as pulmonary congestion causes the “chokes” while vestibular/inner ear injury causes the “staggers.” Neurologic involvement can present with ataxia if due to cerebellar injury. In addition, involvement of cerebral, spinal cord, or peripheral nerves can occur with DCI (8, 51). **Figure 5** demonstrates the creation of an AGE caused by DCI with the rupture of an alveolus and the entrance of alveolar air into the pulmonary venous circulation caused by the decreased external alveolar pressure and resultant expansion and rupture of the alveolus with ascent. DCI often implies the simultaneous presence of gas bubbles in veins and in arteries due to the diffuse nature of gas bubble distribution in blood and tissue. A manifestation of DCI is the AGE associated with pulmonary barotrauma whereby a mixture of air and gas ruptures from the alveoli into the pulmonary venous circulation traveling to the arterial circulation (**Figure 5**). In iatrogenic AAE, a common cause is the entry of air into the pulmonary venous circulation through radiologic procedures with a similar outcome as with the AGE associated with DCI-induced pulmonary barotrauma (**Figure 4**).

### 2.4 Patient positioning: Durant’s maneuver for venous emboli; supine for arterial emboli

Durant’s maneuver in animal studies has been shown to decrease mortality (15, 16). The supposition has been that Durant’s maneuver reduces cerebral embolic air obstruction and relieves the “air lock” on the right side of the heart (2, 4, 15, 62).

Antecedent to Durant’s animal studies, Kent and Blades demonstrated that injection of 1.0 cc of air into the pulmonary vein of 28 dogs was universally fatal. However, when the dogs were injected with the body in a vertical position and the head down, as much as 8–14 cc were tolerated. It was noted that for dogs held in the vertical position, air became trapped in either the left atrium or ventricle and was eventually “forced by ventricular contraction into the aorta.” This 1942 study concerning arterial injection through the pulmonary vein paved the way for Durant’s observations regarding the protective effect of Durant’s maneuver (97).

Durant noted that animals who had been injected with varying volumes of air in the venous circulation survived when placed in the LLD position. This finding led Durant to propose that the immediate assumption of the LLD position with Trendelenburg positioning of the head would allow for the alleviation of air from the pulmonary outflow tract due to the bubble’s positioning in the posterior aspect of the right ventricle where the bubble could be gradually absorbed by the pulmonary circulation. It was proposed that the bubbles would then flow upward into the liver bed due to their buoyancy. Durant’s hypothesis and subsequent research in animals that led to this maneuver was presaged by Van Allen and Hrdina, who in 1929, performed experiments where air was injected into the pulmonary veins. This demonstrated that the animals that had their heads in the Trendelenburg position died due to coronary involvement instead of cerebral involvement since the Trendelenburg positioning of the head avoided cerebral ascension of VAEs. Van Allen and colleagues calculated that animals could withstand pulmonary venous air injections of 0.5 cc/kg when the head was up, 1.5 cc/kg when the head was horizontal, and 3.3 cc/kg when the head was down in the Trendelenburg position. Therefore, Durant’s position was thought to reduce mortality by preventing the bubbles from entering the brain and causing rapid respiratory failure from disrupted CBF to the medulla oblongata or later death by decerebration (6, 15, 16). The explanation for the justification of Durant’s maneuver can be best appreciated by quoting Durant’s 1947 (15) paper with its elegant prose:

“An explanation for the recovery of animals when turned into the left lateral position is to be found in the observations made in the open chest experiments. These have shown that the right ventricle labors against the obstruction of an air trap in its outflow tract when the position of the animal is such as to make this the most superior portion of the right heart. Such would be the case with the animal on his back or on his right side. When turned onto the left side, however, the outflow tract assumes a position inferior to the body of the right ventricle. The air trap then disappears from this, now inferior, position and is presumably churned into a froth which is mixed with the blood in the right ventricular cavity. The obstruction to the circulation is thus relieved and blood can be propelled into the pulmonary vessels by the now unimpeded right ventricular contractions. The froth gradually disappears from the cavity of the right ventricle, and it must be assumed that it has been transported into the lungs with the blood where excretion can take place” (15).

**Figure 6:**
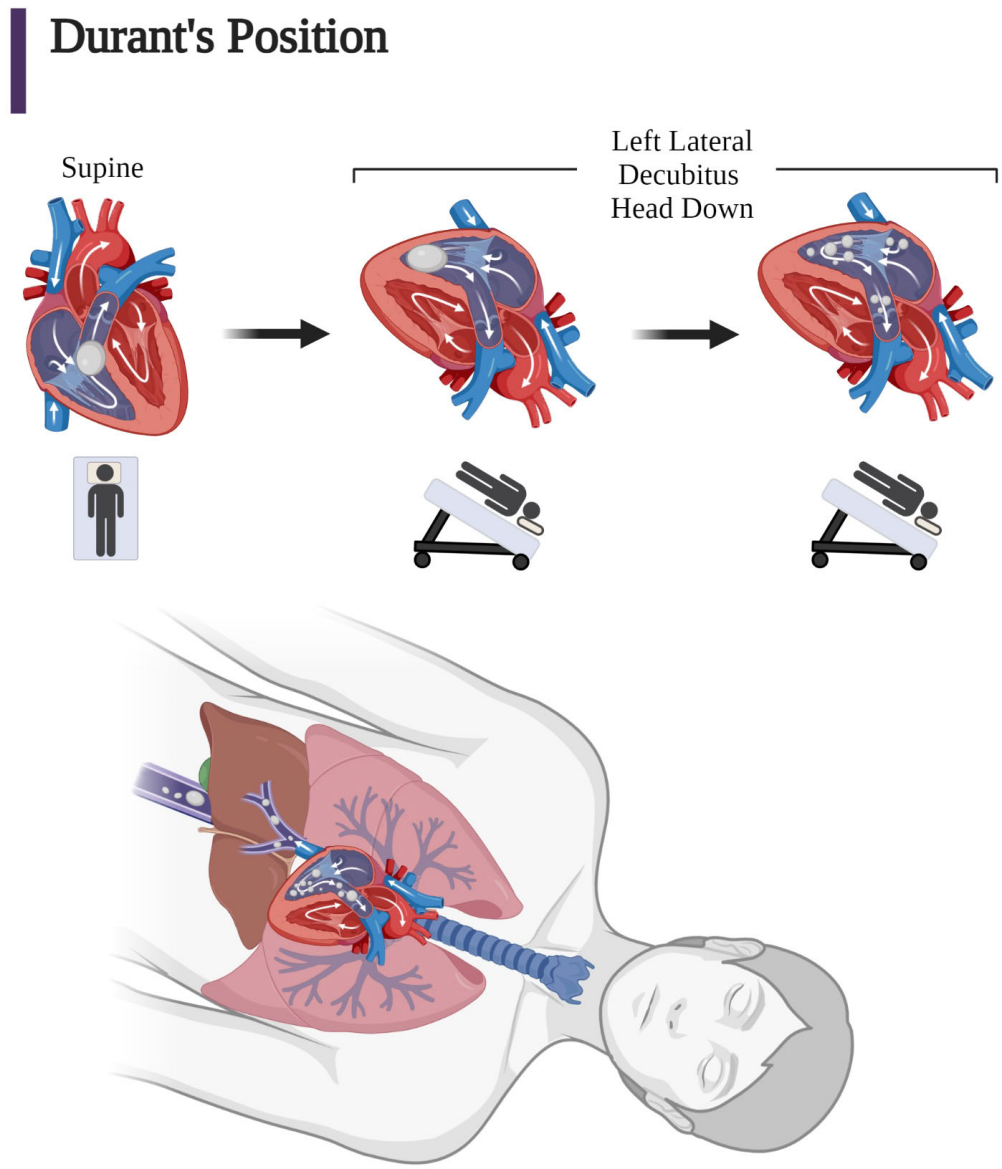
Coronal views of the right ventricle and pulmonary outflow tract based on the open chest observations by Durant after injection of large quantities of air into dogs in the supine position and after Durant’s maneuver. (Left) Supine position: “Air lock” caused by large air bubbles obstructing pulmonary outflow tract in the superior right ventricle. (Middle) Left lateral decubitus position: Immediately after assumption of Durant’s maneuver, air is no longer in the pulmonary outflow tract due to an inferior position in right ventricular outflow tract. (Right) Minutes after assumption of Durant’s maneuver, there is right ventricular compression of the bubble in the inferior aspect of the right ventricle undergoing mechanical induced “frothing” and movement of the smaller bubbles into the pulmonary circulation for absorption. (Bottom) In addition, air in the atrium may also migrate into the vena cava due to the increased mobility of the smaller bubbles, which may result in the microbubbles also migrating upward and distally into the hepatic vein and vena cava circulation (1, 15, 62, 81). (Adapted from Storm 2022) (Created with BioRender.com).

To more clearly explain Durant’s rationale for his maneuver, we rephrase with a modern description the effect of this position observed during the open chest experiments (**Figure 6**) (15). Subsequent to Durant’s paper, it was proposed that some of the smaller bubbles also migrated into the hepatic and inferior vena cava circulation because of their increased mobility. Durant’s explanation has been validated by the observation that external cardiac massage which causes a similar disruption of the large bubble into small bubbles provided equally affected treatment of VAE in dogs when compared to intracardiac aspiration and LLD positioning (62, 81).

In spite of Durant’s direct witnessing of the gradual dissipation of “froth” from the right ventricle with the assumption of the LLD position and the recommendation of Durant’s maneuver as the nitial immediate form of therapy for iatrogenic VAE in the central venous system, the ideal position remains controversial (4, 62, 98). However, assumption of Durant’s position is not beneficial for those patients who have a conversion of a venous to an arterial embolism or those who have direct arterial injection because the bubble is no longer in the venous circulation or right heart. Durant mentioned this when he stated, “The position of the body is much less important therapeutically in arterial air embolism than in the pulmonary form. The head down position does prevent cerebral involvement if the patient is in that position when the embolism occurs, but it does not cause a clearing of the cerebral manifestations if the position is assumed only after the accident has taken place. Furthermore, it does not prevent coronary artery involvement” (15). Therefore, Durant’s maneuver should be limited to those circumstances where there is clear evidence that air has entered the venous system and remains out of arterial circulation where Durant’s maneuver will be deleterious because of the resultant increase in ICP with CAE (4, 13, 15, 19, 62, 68, 82).

## 3 Pathophysiology: the immunothrombotic and endothelial responses to air emboli

Bubble size is a significant determinant for the pathophysiology of air embolisms. It is instructive to speak not just of bubble dimensions but also of vessel size. Arterioles range from 100–300 µm, and minimal thresholds for obstruction have been reported in experimental literature as ranging from 30–250 µm (2, 29, 50).

Small bubbles irritate the vascular wall, which can disrupt the blood-brain barrier (BBB) (99). However, these small bubbles only briefly disrupt the cerebral arteriolar flow as the bubbles are rapidly absorbed (100). There is a correlation between bubble size, blood flow, and brain function. Different thresholds of bubble size that cause relative reduction in CBF have been proposed. For example, bubble size between 30 and 60 µm have been shown to reduce CBF and cerebral function. Other animal studies have demonstrated significant reductions of CBF at 100 µm. It has also been shown that bubble volumes as small as 10 nL may persist for more than 35 minutes, which can significantly impede the delivery of oxygen to areas such as the brain and heart. It has been proposed that a diameter of 250 µm represents a threshold at which CBF is completely impaired (37). At these obstruction threshold values, variable reduction of CBF and cerebral function are likely and may explain the protean manifestations and the delayed response of HBOT (2, 3, 29). Unlike thrombotic emboli, an air embolism may obstruct local blood flow, but if the bubbles disappear during HBOT or through other mechanisms, blood flow will return to normal. However, this return to a normal level of blood flow may only be temporary since subsequent reduction of blood flow can follow (3, 101, 102). It has been shown that the absorption of large air emboli takes several hours. During this time, there may be primary ischemic injury with subsequent diffuse brain edema and an increase in ICP (3, 103).

The BBB may be disrupted because of activation and adhesion of polymorphonuclear leukocytes in the brain (104). Generation of immune mediators induced by complement and coagulation activation by bubbles can provoke resultant thromboinflammatory coagulopathies (32, 35, 44, 104–109). A typical result of air embolism is the migration of air into small arteries with an average diameter range of 30–60 µm (2, 52).

Clinical conditions such as hypovolemia or inspiration, which creates a negative intrathoracic pressure, increase the chances of air entry during procedures involving the venous circulation. The gradient between ambient atmospheric pressure and intravascular CVP is increased by the hemoconcentration of hypovolemia and by inspiration. For patients who are in an upright position or for those who are undergoing IR procedures such as placement of hemodialysis catheters, the CVP is often subatmospheric. These patients are particularly susceptible to a VAE, which may bypass the right heart as the bubbles ascend against venous flow into the cerebral circulation due to their buoyancy (2, 3, 10, 13, 14, 60, 62, 82, 83).

Based on better understanding of the pathophysiologic causes of air embolism emanating from the seminal experimental work by Durant et al. in 1947 (15) and subsequently by others, it was recognized that air emboli commonly cause death through obstruction of outflow from the right ventricle, which has been called “air lock shock” (15, 24, 82, 110). The air emboli in the pulmonary circulation also provoke immediate pulmonary hypertension resulting in right ventricular dilation and right ventricular heart failure due to a release of vasoactive substances such as endothelin-1. The rapid ingress of large volumes of air (> 0.30 cc/kg/min) into the venous circulatory system can overwhelm the air-filtering capacity of the pulmonary vessels, resulting in a myriad of cellular changes. The air embolism effects on the pulmonary vasculature can lead to serious inflammatory changes in the pulmonary vessels, including direct endothelial damage and accumulation of platelets, fibrin, neutrophils, and lipid droplets. Secondary injury resulting from the activation of complement and the release of mediators and free radicals can lead to capillary leakage and eventual noncardiogenic pulmonary edema (3, 4, 111).

Alteration in the resistance of the lung vessels and ventilation- perfusion mismatching can lead to intra-pulmonary right-to-left shunting and increased alveolar dead space with subsequent arterial hypoxia and hypercapnia (1, 2, 4, 15, 24). These occur when the venous air emboli, which are usually filtered in the lung capillaries and expelled through exhalation, overwhelm the filtering capacity of the lung and pass through the lung filter to the arterial circulation (14, 23, 34, 112). Additionally, the presence of a patent foramen ovale, which is present in 10% to 35% of the general population, can also cause these venous air emboli to obstruct myocardial structures (113, 114). The most severe of AAE complications concern obstruction of cerebral and coronary vessels (75). The anatomic obstruction also incites inflammatory and hemostatic responses that initiate platelet activation and thrombus accumulation at the leading and trailing edges of the air bubbles, further obstructing blood flow (29, 115).

### 3.1 Thromboinflammation: hydrodynamics of bubble obstruction

**Figure 7:**
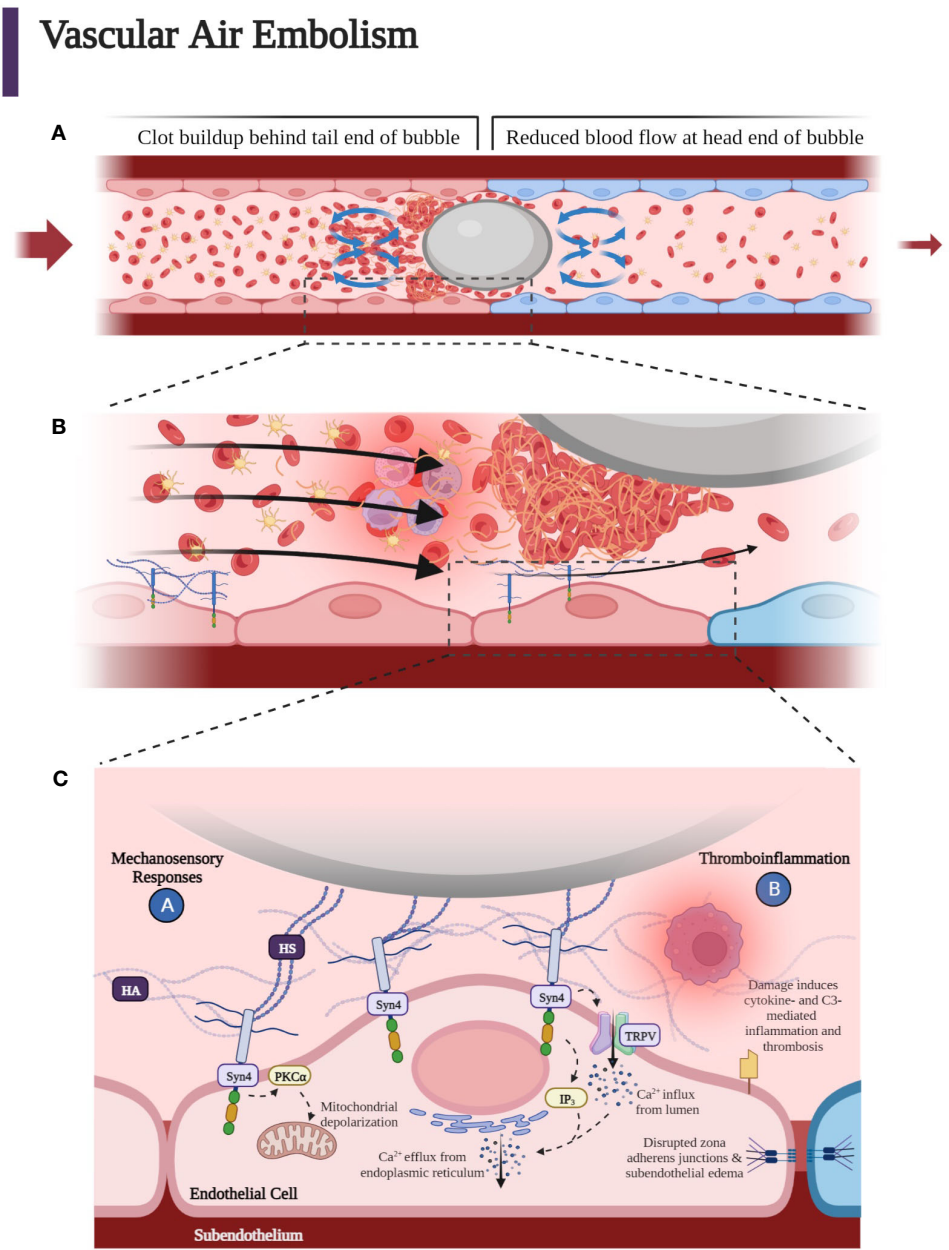
**(A)** As the bubble reaches smaller vessels, it may experience “slug flow” as it deforms to form a Taylor bubble—an elongated bubble that takes up the circumference of the vessel it occupies. The bubble may intermittently stop blood flow when it stops and starts, leading to an increase in pressure that causes the bubble to move again. This process continues so that the bubble stops and starts due to the pulsatile driving pressure behind it. A clot forms at the tail end of the air embolus. Flow recirculation causes fibrinogen, platelets, red blood cells, and white blood cells to accumulate at the tail end of the bubble surface, while the forward flow in front of the bubble moves the cellular components away from the bubble. **(B)** Accumulation of platelets, fibrin, activated white blood cells, and red blood cells around the gas bubble, which with further progression upstream into smaller microvasculature, can lead to hypoxia, penumbra, and terminal ischemia leading to cytotoxic edema and necrosis mediated at the endothelial bubble interface by leukocyte activation, which enhances inflammation causing increased blood viscosity and a reduction in blood flow. Blue endothelial cells represent post-obstructive induced hypoxia. Slug flow can contribute to thromboinflammation due to the buildup of thrombin and fibrin when the bubble slows down. **(C)** Heparan sulfates (HSs) of the endothelial luminal layer have been shown to interact with the air embolism. HS may also interact with the incipient clot at the tail of the embolus as well as with the fibrin, platelets, red blood cells, and activated neutrophils within the lubricant space between the endothelium and bubble. These HS are covalently bound and part of proteoglycans termed syndecans. Syndecans confer mechanosensing capabilities to the endothelium. Syndecan 4 (Syn4) transmits the extracellular shear stress exerted on the HS to the intracellular domain to phosphorylate and activate protein kinase C, which is thought to increase apoptotic signaling through mitochondrial depolarization within the endothelial cell. Inositol triphosphate (IP_3_) signaling molecules open Ca^2+^ channels on the endoplasmic reticulum to cause a transient increase in endothelial cytoplasmic calcium. The increase in cytoplasmic calcium is thought to disrupt adherens junctions, leading to loss of tight junctions between endothelial cells, increased endothelial permeability, and perivascular edema. Moreover, air emboli have been demonstrated to increase tissue factor levels and complement component 3 (C3) activation, leading to thromboinflammation (37, 121). (Created with BioRender.com).

Understanding the fluid dynamics of the moving air bubble confined to a small vessel has shed light on the mechanism of the protean manifestations of air embolisms and lends insight into prevention and treatment. At a theoretical level, recent Monte Carlo simulations of the motion of gas emboli in the cerebral vessels has allowed for modeling deformable gas bubbles, which includes realistic stiction effects and analysis of fluid dynamics, including blood pressure. These simulations show that accumulation of these blockages are partially dependent on blood pressure, which supports the past clinical experimental observation that higher blood pressure can improve the washout of cerebral emboli by more rapidly forcing bubbles through the vasculature in the hypotensive patient (116). Important in the explanation for the differential outcomes of air emboli and blood clot emboli is the theory proposed by Taylor and Bretherton that a bubble in the cerebral vasculature becomes deformed and elongated when it flows in the small microvasculature and that a thin, lubricating film forms around the bubble (117, 118). Hence, the bubble initially keeps flowing without making contact with the endothelium and without adhering to the walls of the vessels due to its slippery, lubricating layer (39). When the bubble encounters a juncture in the vessel, resistance to the progression of the bubble known as “Laplace pressure” further deforms the surface head of the bubble (119). Yet for vessels with a radius greater than 100 µm, the bubble may continue to progress since the Laplace pressure of the bubble is less than 1 kPa, which is insufficient to resist the driving pressure of the bubble (10–15 kPa in small arteries) (29, 120). As the bubble moves into smaller vessels, the deformed bubble surface results in higher pressure within the bubble, which produces drag resistance caused by squeezing of the lubrication film. Yet again, this resistance is not sufficient to prevent progression of the bubble. To further understand the so-called “braking process” of the bubble, recent experiments with microfluidic devices have been employed to define the flow field around the moving bubble and the dysregulation of coagulation caused by this flow field. Microfluidic analyses of air bubbles injected into rabbits have illustrated the location of platelets and red blood cells in various vascular positions of the bubble head, tail, and sides as the bubble forms in the vessel. These experiments have shown further that a clot initiates at the back of the bubble and results in the cessation of the bubble in a small vessel. The recirculation at the tail causes fibrinogen, platelets, red blood cells, and white blood cells to co-locate at the tail end of the bubble surface, while the forward flow in front of the bubble moves the cellular components and fibrinogen away from the bubble (**Figure 7A**). A clot will form when platelets become activated and clotting factors and fibrinogen accumulate at the tail of the bubble (**Figure 7B**). With gradual expansion of the bubble, there is a disruption of the protective lubrication film between the endothelium and the bubble, which has been demonstrated by an optical interference method. Even though microfluidic channels lack an endothelial wall, the concept of “flow recirculation,” which induces thrombus formation that breaks the bubble in microvasculature, is consistent with *in vitro* and *in vivo* experiments (29). Hence, the hypothesis is that the fluid dynamics of so-called “slug flow” and the delicate balance between slug flow and lubrication determine the formation of air emboli in small vessels, and the concept of the Laplace pressure is less important than was once thought. Importantly, these recent studies have affirmed the earlier suggestion that anticoagulation and antiplatelet therapies may retard slug flow caused by the accumulation of fibrin and cellular components at the tail end of the forming air embolism with improvement in the lubrication layer between the air embolism and the endothelium. This mechanism further distinguishes an air embolism from a blood clot embolism because of the lubricant- mediated space, which may allow for continued microperfusion.

Successful treatment of vascular gas embolism with HBOT has been reported as late as 52 hours after development of neurologic symptoms, which contrasts with thromboembolic ischemic cerebrovascular accidents which require thrombolytic intervention within 4.5 hours of symptom onset (53, 122). Recent animal modeling of CAE has elucidated the contrasting pathology. The previously mentioned inhomogeneous distribution of ischemia associated with CAEs can be contrasted with the more definitive and localized ischemia associated with embolic blood clots. The embolic blood clots have been shown using immunofluorescence studies after ferric chloride injury to result in total occlusion within 10 minutes as opposed to the bubble model of arterial occlusion in rabbits, which have a much more prolonged period before ischemia results (123). This may be due to the lubricant space between the deforming bubble and the endothelium, which allows for residual flow around the bubble as well as the *in situ* remodeling of the fibrin and platelet clots at the tail of the bubble as determined by *in vivo* mesenteric arterial emboli in rabbits (29).

In summary, the overall pathophysiologic effect of a gas bubble within the arterial circulation is an obstruction that is mediated by thromboinflammatory changes of the complement and hemostatic systems with resulting vasogenic edema, increased blood viscosity, leukocyte and platelet activation, and increase in ICP—all of which contribute to a decrease of CBF as well as disruption of the BBB (99, 104, 124). This effect of bubbles on cerebral circulation and the BBB leads us to describe the pathophysiological details of the endothelial changes of air-obstructed vessels that cause a transient and sub-lethal reduction of blood flow when compared to thrombotic strokes.

### 3.2 Thromboinflammation: interaction of the endothelial glycocalyx with bubbles, red blood cells, platelets, activated neutrophils, coagulation and complement activation products, and subsequently, the release of cytokines

Maintenance of hemostasis and inflammation were initially thought to be distinct and separate processes involving the coagulation system including tissue factor (TF), coagulation factors, platelets, and endothelium and the complement system, leukocytes, and antibodies. However, the link between coagulation, complement, and inflammatory cells represents a newly appreciated crosstalk between hemostatic and complement pathways, referred to as thromboinflammation (125–130).

While thromboinflammation has been well described in autoimmune disease, infectious disease, and COVID-19-associated coagulopathy (131, 132), there is little research into the thromboinflammatory effects of air emboli in humans. The exact mechanism by which activation of thromboinflammation and the effect of complement inhibition, for example, on thromboinflammation has previously been studied in *in vitro* human whole blood and in *in vivo* animal models (22, 133–136). In an *in vitro* model of air emboli using lepirudin-anticoagulated human whole blood (32) and in an *in vivo* porcine model (35), air emboli activated complement component 3 (C3) and triggered thromboinflammation. Inhibition of C3, but not C5, attenuated inflammatory effects of VAE, although both air emboli and ambient air were found to activate complement and lead to coagulation (36). It has also been shown that air bubbles adhere to and interact with endothelial glycocalyx components such as hyaluronan (HA) and heparan sulfate (HS). In particular, the HS side chains of syndecan 4 (Syn4) are attracted to the bubble’s hydrophobic air-liquid interface, leading to Syn4 being stretched when the bubble moves along. The stretching of the Syn4 causes the transient receptor potential vanilloid (TRPV) Ca2+ channels to open and allow Ca2+ to enter the cell. This interferes with the function of the endothelium, thereby causing capillary leak (30, 37, 121).

Endothelial cells (ECs) respond to hemodynamic stimuli such as altered flow and shear stress *via* electrical and biochemical signals which provoke physiologic and pathological changes at the cellular level. This process is called endothelial mechanotransduction. There has been a discovery of a growing number of highly specific mechanosensors, each with a different function located on the endothelial surface. Examples of these mechanosensors are integrins, stretch-activated channels, platelet endothelial cell adhesion molecules, membrane lipid bilayer, junctional proteins, tyrosine kinase receptors, caveolins, primary cilia, and glycocalyx (30, 40).

Shear stress and altered flow caused by bubbles affect the endothelium at the level of the glycocalyx, which is composed of heparan sulfate proteoglycans (HSPGs) such as Syn1, Syn4, and glypican, as well as glycosaminoglycans (GAGs) such as HS and HA. HS makes up the majority (~50%) of GAGs in the glycocalyx layer and covalently links to HSPGs (137).

The specialized mechanical receptors activated by shear stress and alterations in blood flow at the luminal layer have been studied in several pathological and physiological situations such as in the development of atherosclerosis. This review limits the analysis of mechanical sensors to the glycocalyx where HS acts as a prime mechanosensor attached to the cell by syndecan. HS tethered to cellular syndecan has been shown to play an important role in the pathophysiology not only of bubble-related obstruction to blood flow but embolic clot-related obstruction as well (40, 138–149).

Syndecans have an attachment to the cytoskeleton by virtue of their cytoplasmic tails. Therefore, syndecans can transmit mechanical forces to cellular structures (150–153). In addition, syndecans are linked to G protein receptors and are therefore capable of sensing luminal shear stress and transmitting this change of force into the cell (40, 144, 154, 155). Furthermore, the transmembrane Syn1 core protein is linked to the cytoskeleton and can mediate EC remodeling in response to shear stress (156). This has been demonstrated in the dysregulated response to altered flow in atherosclerotic plaques (157). It has also been noted that Syn4 tethered to glycocalyx HS is crucial for mechanotransduction (158). Therefore, the relationship between the tethered HS and Syn4 is important to understand the crosstalk between the state of luminal blood flow and shear stress and glycocalyx transformation on the cell surface with resultant biochemical and cytoskeletal changes at the cellular level (**Figure 7C**) (159–162).

**Figure 8:**
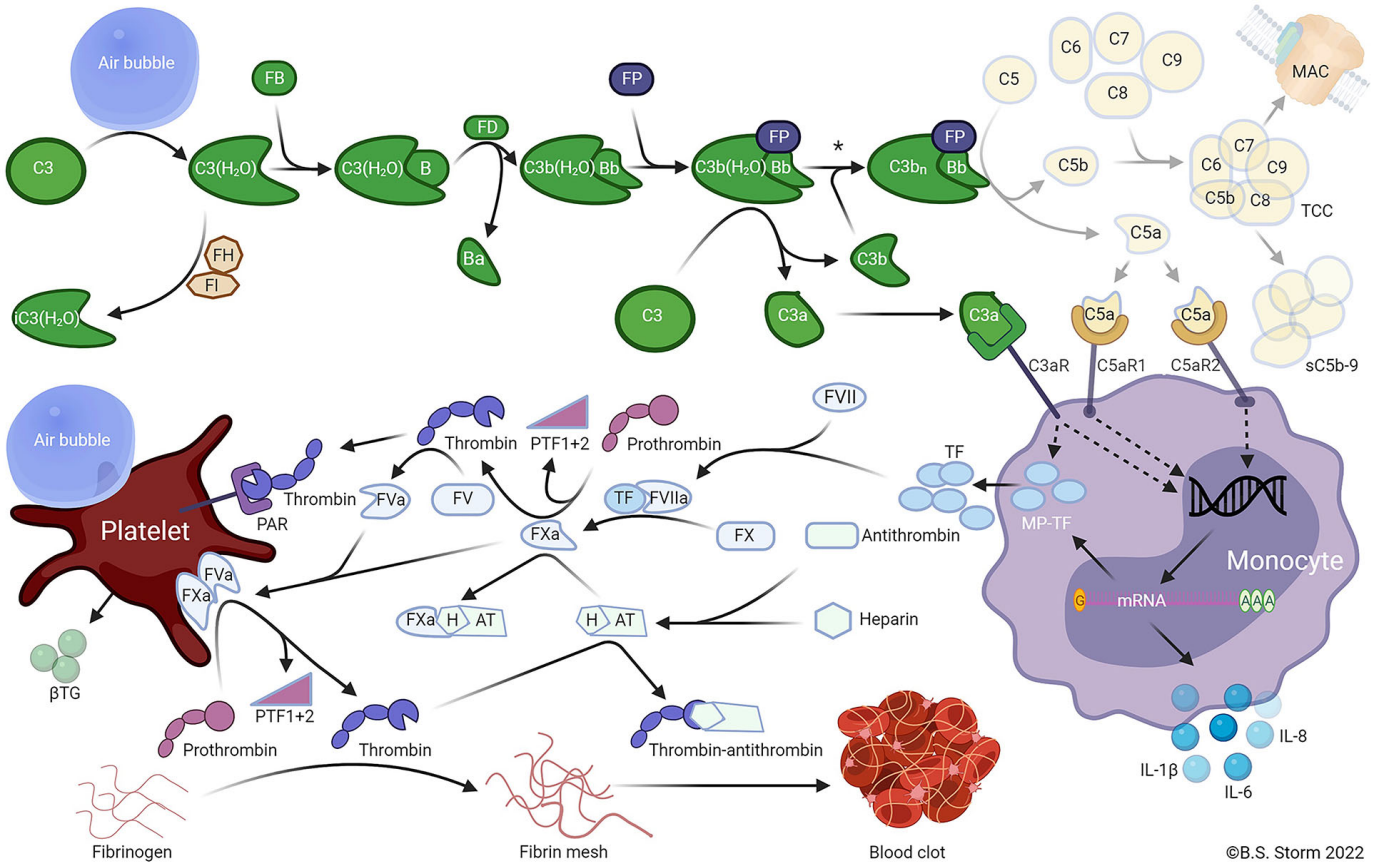
Air emboli activate the alternative pathway and trigger a C3-dependent thromboinflammation. In plasma, C3 undergoes spontaneous slow-rate hydrolysis of the internal thioester to form C3(H_2_O). The C3 hydrolysis is accelerated by contact with foreign surfaces, e.g., an air embolus. C3(H_2_O) is inactivated to iC3(H_2_O) by factor I (FI) in the presence of a cofactor such as factor H (FH). C3(H_2_O) may bind FB (FB) to form C3(H_2_O)B. Catalyzed by factor D (FD), the small Ba fragment is cleaved from C3(H_2_O)B to form the fluid-phase C3 convertase C3(H_2_O)Bb. The C3 convertase is stabilized by properdin (FP) binding to form C3b(H_2_O)BbP. The stabilized C3 convertase cleaves additional C3 molecules to C3b and C3a or forms the C5 convertase C3bnBbP by binding to one or more C3b fragments deposited on foreign surfaces. The C5 convertase then cleaves C5 into C5a and C5b. C5b combines with C6, C7, C8, and C9 to form C5b-9, the terminal complement complex (TCC). TCC may become anchored in cell or bacterial membranes, forming a pore termed the membrane attack complex (MAC), which can cause the lysis of sensitive cells. Alternatively, the TCC may form a soluble complex (sC5b-9) in plasma. The C3a and C5a anaphylatoxins bind receptors for C3a and C5a (C3aR and C5aR1 or C5aR2, respectively) on various cells, including monocytes and granulocytes. The activation of anaphylatoxin receptors on monocytes stimulates *de novo* synthesis and surface expression of tissue factor (TF), extracellular release of microparticles expressing TF (MP-TF), and various inflammatory cytokines, including IL-1β, IL-6, and IL-8. TF binds to coagulation factor VIIa (FVIIa), subsequently activating FX to FXa. FXa catalyzes the cleavage of prothrombin to PTF1 + 2 and thrombin. Thrombin then catalyzes the cleavage of FV to FVa. FVa and FXa combine to form the prothrombinase complex on the surface of activated platelets. The prothrombinase complex cleaves prothrombin into prothrombin fragment 1 + 2 (PTF1 + 2) and thrombin. Platelets can be activated by several mechanisms, including thrombin binding to protease-activated receptors (PAR) 1 and 4, and possibly by direct contact with air emboli, whereby β-thromboglobulin (βTG) and many other mediators are released. Thrombin cleaves fibrinogen to fibrin, which crosslinks and forms a fibrin mesh leading to the formation of a blood clot. The affinity of antithrombin for thrombin is enhanced by binding heparin to form the heparin-antithrombin complex (HAT), which binds to and inactivates FXa. Footnote: The asterisk (*) indicated between the two C3 convertases (upper right) indicates that air emboli-activated C3 is less likely to form an active C5 convertase than is C3 activated by solid substances when C3b is bound to the surface, and the C5a-C5aR axis thus plays only a minor role in air-induced C3-driven thromboinflammation. Note: only key components relevant to the model are included in the figure (36). (Used with Permission from Mollnes et al., 2022) (Created with BioRender.com).

It has been demonstrated that the glycocalyx layer plays an important role in the regulation of bubble-induced Ca2+ transient events through the direct digestion of GAG components. The glycocalyx executes this regulation through deformation forces which are visibly imposed by the bubble that execute protective forces over HA in the underlying cell membrane and interfacial interaction with HS which causes TRPV activation. Thus, HA functions as a cushion-like barrier between the active air-liquid interface along with the underlying glycocalyx components as opposed to functioning as an activating entity (30, 37, 39, 121). The crosstalk between thrombosis and inflammation, which is initiated by the bubbles at the endothelial level is mediated by a complex interaction of hemostatic and inflammatory pathways, in particular, the complement system (**Figure 8**). Bubbles activate the hemostatic pathway initially through platelet activation and the complement pathway through C3 activation. Air emboli have been shown to cause the activation of platelets as well as the release of cytokines, including interleukins (ILs), chemokines, and growth factors (32), and this is central to the pathophysiology of air emboli (9, 22, 30, 32, 33, 37, 135, 136). Air emboli also activate complement *via* the alternative complement pathway as determined by a lepirudin-anticoagulated human whole blood model, resulting in C3 convertase (C3bBbP) formation without simultaneous formation of C5 convertase (9, 32, 36).

The significant effects of complement activation have been studied in pig lung tissue and bear a resemblance to the thromboinflammatory changes in the endothelium found in the hypercoagulable states of sepsis and strokes. Air-mediated C3 activation is associated with the release of proinflammatory cytokines and TF. Air activates the coagulation cascade through a complement-dependent mechanism, as well as a complement- independent mechanism, which also activates platelets. Selective C3 inhibition but not selective C5 inhibition reduces the inflammatory response without a reduction in platelet activation. A porcine *in vivo* experiment has demonstrated that air embolism is associated with thromboinflammation with leukocyte and cytokine release, hemostasis, and C3a and cytokine deposition in the lungs (35). Air embolism provokes a thromboinflammatory response that activates complement C3, leukocytosis, and hemostasis as demonstrated by rotational thromboelastometry (ROTEM) and thrombin-antithrombin. These emboli trigger the synthesis and release of proinflammatory cytokines, IL-1b, IL-6, IL-8, and complement C3a in lung tissue of pigs exposed to air embolism. This reaction confirms the clinical observation that the lungs are an important mediator in the pathophysiologic manifestation of VAE. Analysis by ROTEM and thrombin-antithrombin confirm a significant hypercoagulable state with reductions of clotting times, with increasing clot strength, but without any effect on fibrinolysis as determined by clot lysis index at 30 and 60 minutes. Importantly, venous air embolism in pig lung tissue activates C3 without a corresponding C5 activation and triggers thromboinflammation as manifested by an increase in IL-1b, IL-6, and IL-8 (**Figure 8**) (35, 36).

The thromboinflammatory consequences of air embolism can be compared to those associated with transient ischemic attacks (TIAs) and embolic blood clots. Each can cause hypercoagulopathy and stagnant blood flow along with endothelial injury. Irregular blood flow such as low-shear eddies, recirculation of flow, or loss of shear can cause changes in the shear stress gradient, which can lead to endothelial damage by affecting mechanosensors in ECs (30). In CAE, the resilience of the neuron is manifest by the fact that prolonged symptoms of neurologic impairment can be reversed after long periods of up to 52 hours prior to the inception of HBOT. In addition, the transient improvement with subsequent deterioration followed by recovery bespeaks a similarity of clinical outcome to TIAs. Research has demonstrated that the glycocalyx layer is sensitive to ischemia and reperfusion injury and that markers for endothelial perturbation such as Syn4 and HS demonstrate improvement of the glycocalyx integrity with successful reversal of ischemia with air embolism versus the non-reversal with embolic clots. In TIAs and embolic strokes, there is increased heparanase activity consistent with the higher levels of Syn4 and HS. In contrast to CAE, there is a transient elevation of Syn4 and HS which can be reversed by exogenous surfactant in the rat model (38, 39, 163–165).

The analogy of reversible ischemia with the protean manifestations of CAE reflects the pathophysiologic events which cause endothelial injury in CAE. Unlike ischemic blood clots, microfluidic *in vitro* and animal *in vivo* experiments have demonstrated a lubricating layer between the bubble and endothelium which is analogous to the effect of experimental therapeutic surfactant that has been shown to have an antithrombin effect (29, 45, 165). Surfactants can prevent occlusion of the vessels by rendering the air-liquid interface biologically inert through rapid absorption of the surfactant, which outcompetes other molecules such as proteins for interfacial occupancy, reducing thrombin production by up to 70%. The biological response to air embolism is influenced by biomacromolecular events at the endothelial surface that are linked to transmembrane signaling mechanisms, which cause intracellular responses at the atomic level (Ca2+ entry) and at the cellular level (loss of mitochondrial potential). The result can be the loss of the ability of the EC regulatory inputs into modulation of vascular tone as well as the causation of a procoagulation state due to platelet activation. Thrombin formation is caused by the thromboinflammatory changes brought about by the adhesion interactions between the EC surface and the gas-liquid interface of the bubble. Surfactants can improve all these processes (165). Due to the varying sizes and deformity of the bubble along with temporal dispersion caused by HBOT and transmission of the bubble through arterioles less than 250 µm in size, there are varying levels of obstruction to blood flow that cause varying perturbations of the glycocalyx layer, which acts as a natural surfactant (29, 165). Exogenous surfactant in an experimental mouse model prevents cerebral vascular bubbles from disrupting the mechanosensing mechanism with protection of the tight endothelial junction. Surfactant-treated rats subjected to vascular air embolism had a reduction of syndecan and HS as well as mitigation of selective digestion of glycocalyx GAGs, HA, and HS along with reduced calcium release presumably through direct competition with the endothelial surface layer (38, 39, 165).

Since air bubbles occlude vessels through binding with ECs and diminish air flow by interacting with proteins and initiating thromboinflammation, as mentioned above, attempts to use surfactants to mitigate the obstructive effect of bubbles have been studied. The effects of surfactant on platelet aggregation and thrombin production have shown to have a dose-dependent effect based on the use of platelet aggregometry and flow cytometry measurements. In addition, cellular signaling responses have been affected by various surfactants which demonstrate a reduction on cell lethality caused by bubble contact when surfactant is added to an *in vitro* model. A specific intracellular mechanism by which the bubble interacts with cell surface proteins has shown an increased calcium influx caused by the bubbles which is mediated by cell surface stretch-activated channel and TRPV. This stretch-activated channel, resulting from the mechanical coupling of the bubble and cell, activates an intracellular pathway resulting in a transient intracellular calcium influx from stored calcium in the endoplasmic reticulum which is eliminated by the stretch- activated channel inhibitor, gadolinium, or by TRPV inhibitor ruthenium. In addition, the exposure of this cell to cytochalasin- D, an inhibitor of actin polymerization, also inhibited bubble contact-induced calcium release. Therefore, mechanosensing and stretch-activated channels are necessary for coupling of the extracellular signal of the bubbles surface and cell membrane to the calcium response. This harmful calcium response can be prevented not only by surfactant but also by inhibitors of stretch- activated channels, store-operated calcium channels, and TRPV (38, 43–45, 47–49, 80).

The significance of increased intracellular calcium on endothelial dysfunction caused by bubbles in the clinical setting has been studied and confirmed in rats exposed to DCS. Since bubble formation and endothelial injury have been demonstrated in a DCS rat model and because *in vitro* contact with air bubbles with ECs leads to an increase in cellular calcium and cell damage, it has been proposed that calcium dysregulation contributes to DCI and that a store operated calcium channel blocker would protect the endothelium of rats exposed to DCI. Blockade of store operated calcium channels has been shown to improve DCS in rats by mitigating the effects induced by endothelial activation of platelets, oxidative stress, leukocyte adhesion, and inflammation which lead to endothelial damage and eventual apoptosis. Rats subjected to DCI demonstrated that the gas bubble injuries induced the rapid release of proinflammatory cytokines. The air bubbles induce alveolar macrophages to release many mediators of inflammation such as IL-6, tumor necrosis factor (TNF), a homologue of human IL-8 called cytokine-induced neutrophil chemoattractant-1 (CINC-1), with increased expression of intercellular adhesion molecule-1 (ICAM-1) (12, 38, 49, 80, 166–173).

In addition, disruption of thromboinflammation by air embolism, as measured by quantification of the cytokine gradient which maintains Starling forces and also measured by markers for thrombosis such as microparticle-associated TF is mediated by C3a complement but not by C5 (32, 35). This is yet another example of a difference between the air embolism with its delayed evolution and response to HBOT and embolic blood clots.

In summary, embolic blood clots provoke severe glycocalyx degradation caused by an increased number of proinflammatory cytokines, complement activation as well as a perturbation of the glycocalyx layer as demonstrated by elevation of HS and Syn4 levels. The result is a degradation of the endothelial tight junction with capillary leak as well as mechanical cell death. These severe thromboinflammatory responses are much more extensive than those that occur in vascular air embolism where microvascular flow is maintained by the thin layer of lubricant between the microbubble and the endothelial surface, with less accumulation of fibrin and cellular components such as platelets at the tail of the bubble. Because of these qualitative and quantitative differences in the thromboinflammation in the interaction between the bubble and endothelium, patients with air embolism manifest a significant difference in clinical course than those with thromboembolic blood clots. This difference is mediated by the pathologic finding of a space between air emboli and the endothelium, which causes much less disruption of the bubble and glycocalyx layer. This reduced disruption in the glycocalyx layer is reflected by the prolonged period during which HBOT may be effective following cerebral entry of air and incipient injury, as well as the need for frequent retreatments with HBOT in order to eliminate the cerebral circulation of remnant air emboli. The mediation of the thromboinflammation at the junction of the air embolus and the endothelium may be inhibited by C3a inhibitors and is associated with less elevation of HS and syndecan levels, as well as qualitative differences in cytokine release when compared to embolic blood clots (30, 32, 33, 36, 39, 121, 124, 165).

## 4 Therapy

### 4.1 100% oxygen and hyperbaric oxygen therapy

Treatment of intravascular air embolism, whether venous or arterial, includes breathing 100% oxygen thereby creating a sharp concentration gradient between blood and alveoli, thus accelerating nitrogen washout from the blood through the pulmonary filter. Yet, as has been shown, air emboli can obstruct the flow of blood in capillary structures; therefore, it may be insufficient to eliminate nitrogen bubbles from the air *via* exhaled air. In these cases, HBOT in conjunction with 100% oxygen reduces bubble size, which increases blood flow (20).

HBOT begins with the patient breathing 100% O_2_ and concurrent placement in a hyperbaric chamber. This takes advantage of Boyle’s Law whereby the surface area and volume of the gas bubble are inversely proportional to pressure. Increasing pressure increases the solubility of the gas in the liquid. Thus, exposing the patient to increasing pressure causes the gas bubble to shrink. With this method, arterial pressures of O_2_ can be achieved at greater than 2,000 mmHg. This creates a significant diffusion gradient for O_2_ into the bubble and N_2_ out of the bubble. Nitrogen solubility in plasma is also increased under hyperbaric conditions, driving nitrogen into solution for transport to the lungs and off-gassing (2, 174, 175). In addition, this hyperbaric-mediated hyperoxia permits a great amount of O_2_ to be dissolved in tissues (2, 176). HBOT provides additional benefits related to inflammation by reducing adherence of leukocytes to injured endothelium, preventing cerebral edema with the reduction of the permeability of microvasculature, and improving the BBB (177–179). Due to the beneficial nature of HBOT, an AAE-symptomatic patient should begin HBOT as soon as cardiopulmonary stabilization has been achieved (52, 174, 180, 181). Although most beneficial if initiated early (180), delayed HBOT may still provide significant clinical benefit (53, 58, 103). Based on the hyperbaric literature, significant improvement is still possible even with delays in HBOT initiation greater than 48 hours, and therefore, HBOT should not be denied to a patient who does not arrive within a few hours of the ischemic event. It is with this in mind that HBOT is considered the first-line clinical treatment of choice for AAE (52, 103, 174, 180, 181).

Of significant interest is the well-known phenomenon of transient improvement at maximum atmospheric pressure followed by partial relapse of symptoms once the patient is returned to ambient pressure, which has been described in the DCI and iatrogenic air embolism literature (50, 58, 59).

### 4.2 Enhancing circulating blood volume and increasing arterial pressure

It is important to achieve euvolemia to reduce large pressure gradients from the pulmonary and systemic circulations, thus theoretically decreasing likelihood of VAE conversion to AAE. A correlation between the degree of hemoconcentration and post- treatment residual symptoms has been demonstrated (182–185). It has been shown that patients who have low CVP with the attendant negative pressure gradient between the vein at the entrance of air and the right atrium have an increased risk for VAE. For patients who have CVP monitoring, the ideal CVP should be between 10 and 15 cm of water (4). Animal studies have demonstrated that even a moderate reduction to hematocrit 30% reduces neurologic damage (186). Avoiding glucose-containing solutions is important because it has been shown that even small quantities of glucose may be associated with enhanced lactate production and intracellular acidosis with impaired outcomes (187–190). Dextran solutions are no longer recommended because the theoretical improvements of microcirculation in the anti-sledging effects of dextran observed experimentally have not translated into clinical benefit (190–192). It has been hypothesized that increasing mean arterial pressure (MAP) with the occurrence of an air embolism during cardiopulmonary bypass surgery would increase collateral flow to the affected penumbra and thus decrease the infarct volume and possibly facilitate embolic clearance (3, 193). In a study, rats underwent cardiopulmonary bypass with induced CAEs at three categorized MAPs: high, standard, and low. Improved functional neurological outcomes were seen in rats with a higher induced MAP using whole blood and phenylephrine, but there was no statistical difference in infarct volume. The inconsistency between improved neurological function and infarct volume is thought to be due to air embolism’s ability to induce thromboinflammation. Increasing MAP may help ameliorate the effects of thromboinflammation with embolic clearance (193).

### 4.3 Mechanical removal of air with risk stratification of diagnosis and treatment

Mechanical removal of air requires anticipation and preparation in the operating theater, intensive care unit, or IR unit whenever there is the possibility of the entrance of large quantities of air into the venous circulation. At the appearance of symptoms, withdrawal of air should be attempted if a catheter is in place to prevent the migration of bubbles. Examples of those situations include the development of an unexplained sudden reduction of intraoperative end-tidal carbon dioxide (ETCO_2_) or the development hypotension particularly in cases which are performed in reverse Trendelenburg or in those cases where venous vasculature is exposed to atmospheric pressure. Of particular importance is the development of dyspnea or hypotension in patients who have recently had central venous lines placed or removed. Anticipation of the need to remove air is a function of the likelihood of the risk of an air or gas embolism during procedures. If the procedure is performed in a fluoroscopic suite, the diagnosis may be established radiographically, with air seen in the pulmonary outflow tract and pulmonary arteries. **Table 1** provides an approximation of the relative risk of the development of air or gas embolism as a function of the type of surgery or procedure (4). This table is emphasized because the immediate diagnosis of air embolism is essential to determine the need for rapid mechanical removal of air, and placement of the patient with Durant’s maneuver depends on an understanding of the relative risks of developing VAE and AAE for each operation. Those procedures, which are done in the sitting position, are most likely to cause VAEs and will require immediate treatment with mechanical removal of air and assumption of Durant’s maneuver (4, 62, 68–70).

**Table 1:** Risk assessment for iatrogenic venous air embolism in procedures from high-risk to low-risk operations (4, 13, 82).

High-Risk	Medium-Risk	Low-Risk
Sitting position craniotomy	Spinal fusion	Peripheral Nerve Procedures
Posterior fossa/neck surgery	Cervical laminectomy	Anterior Neck Operations
Laparoscopic procedures	Prostatectomy	Burr hole neurosurgery
Total hip arthroplasty	Gastrointestinal endoscopy	Vaginal procedures
Cesarean delivery	Contrast radiography	Hepatic surgery
Central venous access placement or removal	Blood infusion	
Craniosynostosis repair	Cardiac surgery/cardiopulmonary bypass, extracorporeal membrane oxygenation	

When possible, placing the patients in a Trendelenburg position should be a priority to increase the CVP and therefore to reduce the pressure gradient. In addition, during the placement of venous catheters it is important, if possible, to ensure that these patients are adequately hydrated to increase the CVP. The avoidance of forced inspiration and the consequent negative intrathoracic pressure is essential when placing central venous catheters. The reduced risk of air embolism is enhanced by avoiding a short subcutaneous path to the jugular vein. In the absence of guide wires, there should be careful attention to occlusion of the hub with the thumb as well as adherence to flushing all central line catheters completely before placement. Practitioners should have in-depth understanding of the valve mechanisms and the types of catheters they insert with particular attention to the so-called “peel away” sheath, intravenous port, self- sealing valves, and Luer lock connectors (4). When placing hemodialysis catheters through a “peel away” sheath, it is essential to be sure both lumens of the catheters are clamped before introducing the catheter. During the removal of catheters, it is important that the catheter site remain at the level of the heart with the assumption of a Trendelenburg position, and if possible, the patient should be instructed to perform a Valsalva maneuver (4, 194).

Precordial and transesophageal echocardiography during the intraoperative period allows for early detection of air in the heart. With the recognition of cardiac air, particularly with associated hypotension, shock, and cardiac arrest, immediate evacuation through a central line is advocated. Similar attention to this complication is important when placing or removing hemodialysis catheters, central venous catheters, or other intravascular devices. Placing the patients in the Trendelenburg position when possible is important, particularly in cases where the patient is sitting. Immediate initiation of 100% oxygen will both optimize tissue oxygenation and provide a gradient for more efficient off-gassing of nitrogen (4, 68).

High success rates for air aspiration have been demonstrated with multi-lumen Swan-Ganz catheters or specific devices with large diameter such as > 5 French catheters. If mechanical aspirations are attempted, it has been suggested the catheter be positioned 2 cm distal to the superior vena cava from the atrial junction. The volume of air withdrawn has been suggested to be between 15 and 20 cc. However, because of the “frothing” noted by Durant with the use of Durant’s maneuver, a mixture of air and blood will be withdrawn until the signs and symptoms improve. Therefore, the quantity of air and blood withdrawn is a clinical decision (4, 195–201).

## 5 Additional potential treatments for air embolism

### 5.1 Use of anti-inflammatory agents against the effects of air embolism

It has been well established and previously mentioned that pulmonary air embolisms can induce alveolar macrophages to release inflammatory factors such as IL-6, TNF, and CINC-1. These factors contribute to acute lung injury induced by air embolisms. This has led to the idea of using anti-inflammatory drugs as a potential treatment vector. In one study, rats pre-treated with baicalin showed attenuation of TNF, CINC-1, and significantly improved histological appearance. Free radicals, proinflammatory cytokines, and nuclear factor kappa B were significantly suppressed (166). However, the role of anti-inflammatory drugs in treatment of patients with air embolism is not fully elucidated, and routine use is not generally recommended.

### 5.2 Use of surfactant to mitigate bubble-induced occlusion *via* thromboinflammation

In section 4.2, we described the *in vitro* and *in vivo* data demonstrating the effects of surfactant in treatment of bubble-induced vascular obstruction. Prompted by the problem of air embolism associated with the use of bubble pump oxygenators in the early days of open left heart surgery, there was interest in the use of surface tension-reducing substances, also known as surfactants, to reduce the incidence of air embolism in these cases (202). Subsequently, it was noted that simultaneous injection of surfactant and air into coronary arteries was able to reduce lethality by approximately 50%. This has led to a search for biocompatible surfactants, which could be used in the operative theater (203). Considerable interest remains in this area since the incidence of postoperative central nervous system pathology has been attributed to the effect of microbubbles on the cerebral vasculature for patients undergoing extracorporeal membrane oxygenation (ECMO) or cardiopulmonary bypass (37, 165, 176, 204). Perfluorocarbons (PFCs) have been used as an experimental substitute for blood, in intraocular injection, to treat DCS and avoid iatrogenic CAE in both human and animal experiments and most recently, thoracic endovascular aortic repair (TEVAR) (205–209). Recent studies regarding the surfactant perflubron in patients following cardiac and orthopedic surgery have revealed conflicting data, and therefore, its use remains experimental in humans (210).

### 5.3 Anticoagulation, antiplatelet, and anticomplement therapy

Earlier studies have demonstrated benefits of anticoagulation and antiplatelet therapy in animal models, but few clinical reports exist in the literature. Yet it is logical to think that antiplatelet therapy would represent a safer option, which may be the object of future studies in humans (211). Previous studies with pharmacologic intervention against bubble-induced platelet aggregation in a rat model of DCI have demonstrated that specific ADP receptor antagonists reduced post-decompression platelet consumption. However, anti-arachidonic acid treatment, which inhibits the thromboxane A2 pathway, does not reduce post-decompression platelet consumption. These findings confirm earlier demonstration of the relative importance of ADP dysfunction of platelets associated with the endothelial injury of trauma-induced coagulopathy (115, 212).

A deeper understanding of the relationship of thrombosis in inflammation at the level of the injured cerebral endothelium may compel clinicians to begin trials of adjunctive anticoagulant and antiplatelet therapies in patients with air bubble-related injury. As has been seen with the use of heparin in treating patients with thromboemboli associated with COVID-19 pneumonia, many areas along the thromboinflammatory pathway are affected by heparin. Current evidence suggests that heparin is ineffective in the treatment of air embolism unless it is administered immediately. Due to the intracranial bleeding risk associated with heparin, preventive anticoagulation would not be advocated for CAE unless it was already required for carotid or cardiac surgery. However, given the mechanisms of thromboinflammation that cause endothelial and cell injury following CAE, inhibition of the thromboinflammatory pathways could be a therapeutic benefit. For example, selective thrombin inhibition (213, 214), inhibition of leukocyte adherence and action (215, 216), or blockade of C3 have been proposed as possible therapeutic modalities (32), and future research should examine the role of these agents in the treatment of air embolism.

Thromboinflammatory mechanisms may partially mediate neurological injury caused by CAE, which suggests inhibition of thromboinflammatory pathways as a possible treatment. Additionally, while C3 inhibition *via* compstatin was important for delineating this unique pathophysiological mechanism *in vitro*, a C5 inhibitor was largely ineffective, highlighting C3 as a possible target for treatment (32, 33, 35, 36).

### 5.4 Reduction of bubble-induced hypercoagulability with membrane stabilizing anesthetics

Preclinical studies have demonstrated that medications such as lidocaine, ketamine, and magnesium sulfate may inhibit platelets as well as hypercoagulability. It has been noted in relation to patients with DCI who have CAE that lidocaine is of benefit to these patients possibly by its action on membrane stability (115, 217–221). Recent thromboelastographic (TEG) studies in rats have demonstrated that the hypercoagulable changes induced by bubbles in a rat model can be mitigated using mexiletine, which is a class I-b local anesthetic and lidocaine analog. Because this medication can be taken orally it has been proposed that it would have increased clinical use in the setting of CAE. In rats subjected to experimental VAEs, there was a significant reduction of TEG reaction time (R) and kinetics (K) parameters with a concomitant increase in the alpha-angle and maximal amplitude parameters all indicating a hypercoagulable state. In mexiletine treated animals, there was a prevention of the maximal amplitude increase caused by VAE and a corresponding correction of the R and K times at 24 hours. Therefore, it was proposed that the hypercoagulable state associated with VAEs could be mitigated using this oral lidocaine analog. This thromboelastographic analysis confirms that the presence of an air bubble interface can activate primary hemostasis by augmenting the adhesion and aggregation of platelets as well as secondary hemostasis by increasing the activity of coagulation of the intrinsic and extrinsic coagulation factors at the site of endothelial injury (43, 115, 133, 222–224). However, lidocaine may have cardiotoxic side effects and is not routinely administered to patients with air embolism (225).

### 5.5 Endothelial targeting protection therapy

Endothelial protection agents such as simvastatin may be of benefit in treating DCS. Since endothelial dysfunction has been described in DCS and correlated with experimental bubble formation in a rat model it has been proposed that an endothelial protected agent able to reduce vascular permeability, venous congestion, free radical production, and increase vascular elasticity and tension may be a benefit in offering endothelial protection. In an experimental model, escin, an endothelial protective extract from the Aesculus hippocastanum plant, was able to improve the thrombocytopenia and endothelial dysfunction as well as the oxidative and inflammatory responses of swine exposed to simulated diving. Specifically, escin significantly reduced thrombocytopenia as well as reducing the levels of cytokines IL-1b and IL-6. Finally, escin was able to reduce the bubble induced increase of serum ICAM-1, endothelin-1, and methane dicarboxylic aldehyde as tested by ELISA (226). However, simvastatin is not routinely recommended for the patients with air embolism.

### 5.6 Massive venous air embolism: closed- chest cardiac massage as a therapeutic maneuver and aggressive repeat manual pulmonary recruitment maneuver with pure oxygen in the operating theatre

For those patients presenting with massive VAE that results in cardiac standstill, cardiopulmonary resuscitation with chest compressions and defibrillation if there is ventricular fibrillation present must be initiated immediately (227). Even in those situations without the need of cardiopulmonary resuscitation, for patients in shock, there is a rationale behind the closed-chest massage of the heart, which forces air out of the pulmonary outflow tract into the smaller pulmonary vessels. Animal studies have suggested that external cardiac massage is as effective as the mechanical aspiration of air or the LLD position in preventing death caused by a rapid dose of 15 cc/kg of air in dogs. This study confirmed the importance of “frothing” as noted by Durant; however, the “frothing” of the blood in the right axis of the heart is accomplished by external cardiac massage. This has led to recent reports of the use of repeat manual pulmonary recruitment maneuver combined with pure oxygen to improve hemodynamics of a patient who had suffered a large iatrogenic and intraoperative VAE who did not respond to standard therapy (81, 110).

**Figure 9:**
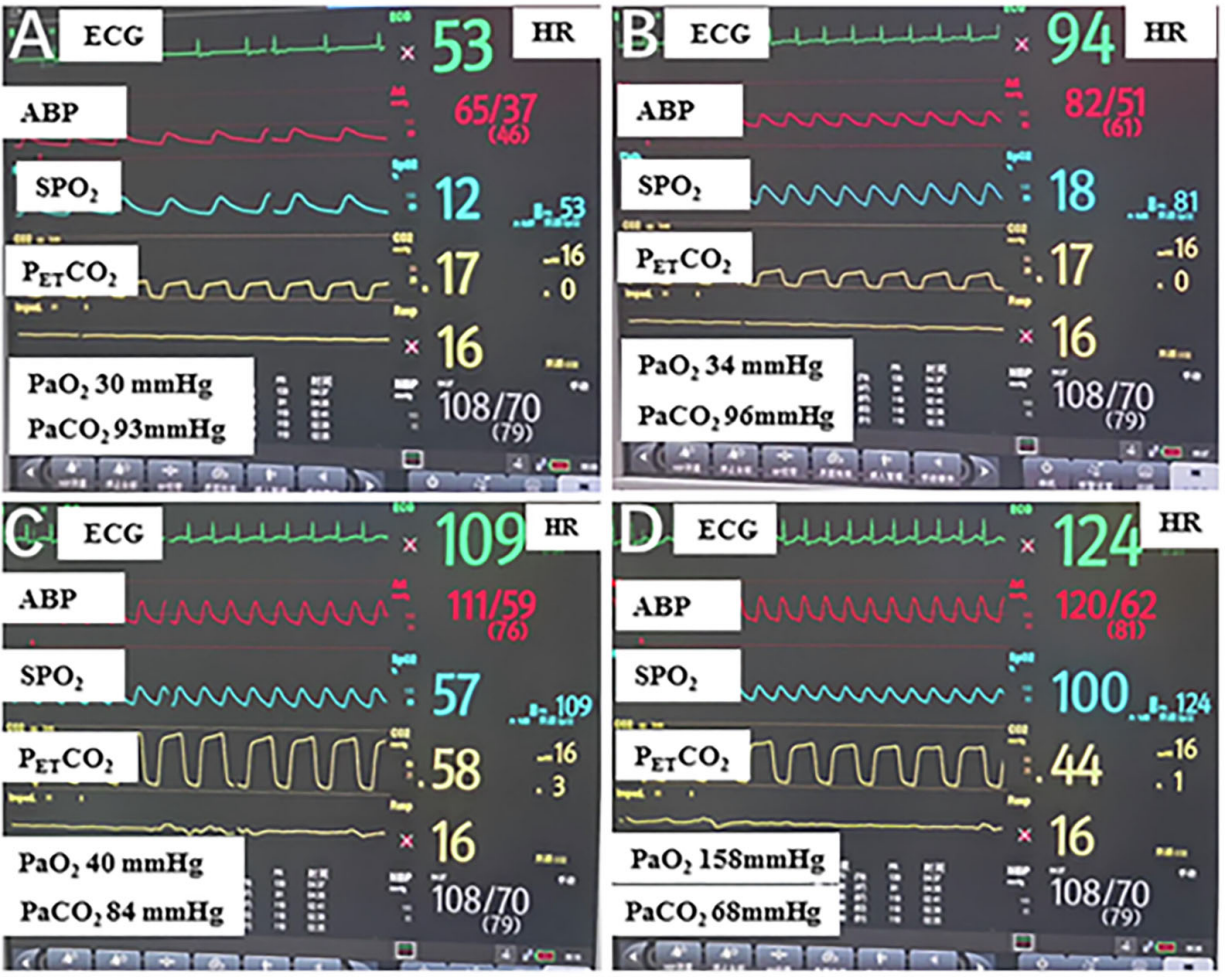
The changes in a patient’s vital signs during operation after treatment. **(A)** Patient’s vital signs when venous air embolism was considered; **(B)** Patient’s vital signs after Durant’s maneuver, sealing the operative field with saline, using pure oxygen, fluid resuscitation, and hemodynamics support. **(C)** Patient’s vital signs after an aggressive manual pulmonary recruitment maneuver was repeated for five minutes. **(D)** Patient’s vital signs after an aggressive pulmonary recruitment maneuver repeated for 10 minutes. ECG, Electrocardiograph; HR, heart rate; ABP, arterial blood pressure; SPO_2_, oxygen saturation; P_ET_CO_2_, end-tidal carbon dioxide partial pressure; PaO_2_, arterial oxygen pressure; PaCO_2_, arterial carbon dioxide pressure (110). (Used with Permission from Zheng et al., 2022).

Based on a clinical case, a novel approach for patients on mechanical ventilation who develop VAE has been recently described as aggressive repeat manual pulmonary recruitment maneuver with pure oxygen, which is a new ventilation strategy designed to ameliorate respiratory mechanics, thereby increasing pulmonary oxygen exchange and arterial oxygenation. In addition, this technique has been proposed to cause right ventricular contraction with the resultant reduction in size of the air embolism thereby facilitating movement of the obstructing air bubble from the pulmonary outflow tract. This treatment has been proposed for those patients who suffer iatrogenic, intraoperative VAE who do not respond to standard treatment (**Figure 9**). Large lung volumes with aggressive pulmonary recruitment maneuvers compress the compliant right heart, forcing the air embolism into smaller bubbles more amenable to traversing the pulmonary outflow tract into the smaller pulmonary vessels. Secondly, the release of the lung before lung hyperinflation benefits venous return. With subsequent hyperinflation, increased blood flow out of the right ventricle facilitates movement of air out of the right ventricular outflow tract. Currently, animal studies have been proposed with possible clinical trials to follow (110).

Finally, for those patients who do not respond to aspiration of air, placement in Durant’s maneuver, or external cardiac massage, it has been recommended that thoracotomy with open cardiac massage may reduce the size of the bubbles so that they may transverse into pulmonary circulation and be filtered (228).

However, as noted by Durant and confirmed by others in a porcine model of VAE, thoracotomy with pericardiotomy facilitated the transpulmonary passage of intravenously infused air and greatly reduced the threshold for when air reached the systemic circulation through the lungs, potentially causing end- organ ischemia and death (15, 34).

## 6 Prevention and early diagnosis of vascular air embolism: clinical vigilance and the use of multi- monitoring technologies

In the operating theater, preventive measures such as avoiding surgical field above heart level, adequate hydration, avoidance of nitrous oxide, and scrupulous placement and removal of catheters should be taken. For those clinical situations where it is not possible to have the patient in a Trendelenburg position for the duration of a procedure, it has been recommended that the legs be raised by placing a pillow under the knees to increase venous return and therefore right atrial pressure during insertion of guide wire or catheter. For surgical positioning, the head up position places the patient at greater risk for CAE, but the air embolism may even occur during shoulder surgeries and other surgical procedures in proximity to the head and neck (4, 13, 229, 230). For central venous catheter placement, preventing air embolism occurs by clearing the central line of air prior to insertion, the use of the head down position and Valsalva maneuver during insertion and removal, the use of screw-on connections secured with tape, and the avoidance of hemostats that can crack the hub causing an open system, the use of air-occlusive dressing during central venous catheter use and after removal (231). Until recently, checklists for the prevention of air embolism have not existed, and it has been proposed that an air embolism be listed as a “never event” by the National Quality Forum, which raises consciousness about the preventability of iatrogenic air embolism (14, 232).

Monitoring for acute changes in vital signs such as blood pressure, respiratory/cardiac rate and rhythm, electrocardiogram waves, oxygen saturation, and ETCO_2_ are important for early detection. Doppler ultrasound has been shown to be a sensitive monitoring system for air embolism with detection of as little as 0.25 cc of air. It should be mentioned that the high sensitivity associated with the transesophageal echocardiography can produce “false alarms” with the unnecessary intervention due to its detection of nearly any amount of air in circulation, which is controversial (4, 229).

Of importance, end-tidal nitrogen (ETN_2_) has been shown to be the most sensitive gas for detecting air embolism. Comparing ETCO_2_ with ETN_2_, it was found that monitoring N_2_ showed changes 30–90 seconds earlier than CO_2_ monitoring for air embolisms. ETN2 is limited in availability and not useful when nitrous oxide in oxygen is used in anesthesia (4).

The use of air detectors in rapid fluid infusion systems and extracorporeal circuits such as those seen in ECMO, dialysis, and plasmapheresis machines are integral to detection. Recent application of the liquid surfactant PFC as a flushing agent with CO_2_ to reduce the amount of gas released during stent-graft deployment in TEVAR has demonstrated a significant reduction in the volume of gas released during deployment of the tubular thoracic stent-graft based on the high solubility of the CO_2_ in PFC. PFC as a surfactant provides a reduced surface tension and diminishes the sticking of air in small compartments. This factor may cause the reduced amount of air released during stent-graft deployment when compared to a saline solution (205, 207). Recent research combining artificial intelligence with Doppler ultrasound recordings for vascular air embolism detection and grading suggests promise for combining traditional and emerging technologies for improved diagnostic and bedside care in the area of air embolism (4, 233). Antiquated yet of paramount importance, simple caution and alertness may allow surgeons and anesthesiologists to see or hear air being aspirated into circulation during the case while simultaneously reviewing the multi- technological monitoring devices for confirmation of the presence of a vascular air embolism (4).

## 7 Conclusions

Iatrogenic vascular air embolism is a relatively infrequent event but is associated with significant morbidity and mortality. These emboli can arise in many surgical settings. However, more recently, endoscopy, hemodialysis, thoracentesis, tissue biopsy, angiography, and central and peripheral venous access and removal have overtaken surgery and trauma as significant causes of vascular air embolism. The foundation for understanding the pathophysiology of air embolism lies in an appreciation of the crosstalk between thrombosis and inflammation, where complement seems to play a significant role, within the lumen of the blood vessel and between the bubble itself and the endothelium.

## Author contributions

Conceptualization: PM, EM, HM, CMB, JM, HK, BS, and MW; Original Draft Preparation: PM, CMB, EM, HM, HK, JM, BS, SC, MA-F, SJT, SA, and MW; Writing—Review and Editing: PM, EM, HM, CMB, MA, SC, MA-F, SJT, JL, CWB, CM, MP, CT, DR, GK, BT, DY, SGT, DS, SSP, SA, AT, JM, SVP, DZ, BW, DR, PLM, CS, JA, RG, RM, HK, DF, SL, TM, EN, BS, and MW; Visualization: PM, CMB, SC, MA-F, SJT, SA, TM, EN, BS, and MW; Supervision: MW. All authors contributed to the article and approved the submitted version.

## Glossary

**Table:**

AAE	Arterial Air Embolism
ABP	Arterial Blood Pressure
AGE	Arterial Gas Embolism
BBB	Blood-Brain Barrier
CAE	Cerebral Air Embolism
CBF	Cerebral Blood Flow
CINC-1	Cytokine-Induced Neutrophil Chemoattractant-1
CVP	Central Venous Pressure
CX	Complement Component X
DCI	Decompression Illness
DCS	Decompression Sickness
EC	Endothelial Cell
ECMO	Extracorporeal Membrane Oxygenation
ETCO_2_	End-Tidal Carbon Dioxide
ETN_2_	End-Tidal Nitrogen
GAG	Glycosaminoglycan
HA	Hyaluronan
HBOT	Hyperbaric Oxygen Therapy
HR	Heart Rate
HS	Heparan Sulfate
HSPG	Heparan Sulfate Proteoglycan
ICAM-1	Intercellular Adhesion Molecule-1
ICP	Intracranial Pressure
IL	Interleukin
IP_3_	Inositol Triphosphate
IR	Interventional Radiology
K	Kinetics
LLD	Left Lateral Decubitus
MP	Microparticle
PaCO_2_	Arterial Carbon Dioxide Pressure
PaO_2_	Arterial Oxygen Pressure
P_ET_CO_2_	End-Tidal Carbon Dioxide Partial Pressure
PFC	Perfluorocarbon
R	Reaction Time
ROTEM	Rotational Thromboelastometry
SPO_2_	Oxygen Saturation
SynX	Syndecan X
TEG	Thromboelastogram
TEVAR	Thoracic Endovascular Aortic Repair
TF	Tissue Factor
TIA	Transient Ischemic Attack
TNF	Tumor Necrosis Factor
TRPV	Transient Receptor Potential Vanilloid
VAE	Venous Air Embolism
VSD	Ventricular Septal Defect
