# Corrigendum: Iatrogenic air embolism: pathoanatomy, thromboinflammation, endotheliopathy, and therapies

**DOI:** 10.3389/fimmu.2024.1378003

**Published:** 2024-02-06

**Authors:** Phillip L. Marsh, Ernest E. Moore, Hunter B. Moore, Connor M. Bunch, Michael Aboukhaled, Shaun M. Condon, Mahmoud D. Al-Fadhl, Samuel J. Thomas, John R. Larson, Charles W. Bower, Craig B. Miller, Michelle L. Pearson, Christopher L. Twilling, David W. Reser, George S. Kim, Brittany M. Troyer, Doyle Yeager, Scott G. Thomas, Daniel P. Srikureja, Shivani S. Patel, Sofía L. Añón, Anthony V. Thomas, Joseph B. Miller, David E. Van Ryn, Saagar V. Pamulapati, Devin Zimmerman, Byars Wells, Peter L. Martin, Christopher W. Seder, John G. Aversa, Ryan B. Greene, Robert J. March, Hau C. Kwaan, Daniel H. Fulkerson, Stefani A. Vande Lune, Tom E. Mollnes, Erik W. Nielsen, Benjamin S. Storm, Mark M. Walsh

**Affiliations:** ^1^ Department of Emergency Medicine, Saint Joseph Regional Medical Center, Mishawaka, IN, United States; ^2^ Department of Surgery, Ernest E. Moore Shock Trauma Center at Denver Health and University of Colorado Health Sciences Center, Denver, CO, United States; ^3^ University of Colorado Health Transplant Surgery - Anschutz Medical Campus, Aurora, CO, United States; ^4^ Department of Emergency Medicine, Henry Ford Hospital, Detroit, MI, United States; ^5^ Indiana University School of Medicine, South Bend, IN, United States; ^6^ Department of Emergency Medicine, Goshen Health, Goshen, IN, United States; ^7^ Department of Family Medicine, Saint Joseph Health System, Mishawaka, IN, United States; ^8^ Department of Trauma & Surgical Research Services, South Bend, IN, United States; ^9^ Department of Emergency Medicine, Beacon Health System, Elkhart, IN, United States; ^10^ Department of Internal Medicine, Mercy Health Internal Medicine Residency Program, Rockford, IL, United States; ^11^ Department of Cardiovascular and Thoracic Surgery, RUSH Medical College, Chicago, IL, United States; ^12^ Division of Hematology and Oncology, Department of Medicine, Northwestern University, Chicago, IL, United States; ^13^ Department of Emergency Medicine, Naval Medical Center Portsmouth, Portsmouth, VA, United States; ^14^ Research Laboratory, Nordland Hospital, Bodø, Norway; ^15^ Faculty of Medicine, Institute of Clinical Medicine, University of Oslo, Oslo, Norway; ^16^ Department of Immunology, Oslo University Hospital, University of Oslo, Oslo, Norway; ^17^ Department of Anesthesia and Intensive Care Medicine, Surgical Clinic, Nordland Hospital, Bodø, Norway; ^18^ Institute of Clinical Medicine, University of Tromsø, Tromsø, Norway; ^19^ Faculty of Nursing and Health Sciences, Nord University, Bodø, Norway

**Keywords:** air embolism, decompression sickness, hyperbaric oxygenation, thromboinflammation, microbubbles, arterioles

In the published article, there was a recurring error regarding the units of measurement for bubble diameter and radius. Ten (10) instances of the correct micrometer (μm) were erroneously changed to millimeter (mm) in the proof corrections process (during which a case study was removed from the paper). In these 10 instances when “mm” appears, “μm” should instead appear.

1. A correction has been made to **2 Pathoanatomy and importance of patient positioning**, paragraph 2. This sentence previously stated:

“In canine experiments, bubbles that were too small to get trapped (smaller than 22 mm) could enter directly into arterial circulations and provoke inflammation as they passed through the pulmonary circulation (65).”

The corrected sentence appears below:

“In canine experiments, bubbles that were too small to get trapped (smaller than 22 μm) could enter directly into arterial circulations and provoke inflammation as they passed through the pulmonary circulation (65).”

2. A correction has been made to **2.2.2 Direct arterial air embolism**, paragraph 2. This sentence previously stated:

“Durant’s initial findings in canines have been supported by subsequent studies on anesthetized swine wherein air bubbles with diameters of 150 mm and volumes of 0.002 cc/kg of body weight were injected into coronary circulation and caused severe life-threatening arrhythmias and signs of global cardiac dysfunction (3, 85, 86).”

The corrected sentence appears below:

“Durant’s initial findings in canines have been supported by subsequent studies on anesthetized swine wherein air bubbles with diameters of 150 μm and volumes of 0.002 cc/kg of body weight were injected into coronary circulation and caused severe life-threatening arrhythmias and signs of global cardiac dysfunction (3, 85, 86).”

3/4. A correction has been made to **3 Pathophysiology: the immunothrombotic and endothelial responses to air emboli**, paragraph 1. This sentence previously stated:

“Arterioles range from 100–300 mm, and minimal thresholds for obstruction have been reported in experimental literature as ranging from 30–250 mm (2, 29, 50).”

The corrected sentence appears below:

“Arterioles range from 100–300 μm, and minimal thresholds for obstruction have been reported in experimental literature as ranging from 30–250 μm (2, 29, 50).”

5. A correction has been made to **3 Pathophysiology: the immunothrombotic and endothelial responses to air emboli**, paragraph 2. The sentence previously stated:

“For example, bubble size between 30 and 60 mm have been shown to reduce CBF and cerebral function.”

The corrected sentence appears below:

“For example, bubble sizes between 30 and 60 μm have been shown to reduce CBF and cerebral function.”

6. A correction has been made to **3 Pathophysiology: the immunothrombotic and endothelial responses to air emboli**, paragraph 2. The sentence previously stated:

“Other animal studies have demonstrated significant reductions of CBF at 100 mm.”

The corrected sentence appears below:

“Other animal studies have demonstrated significant reductions of CBF at 100 μm.”

7. A correction has been made to **3 Pathophysiology: the immunothrombotic and endothelial responses to air emboli**, paragraph 2. The sentence previously stated:

“It has been proposed that a diameter of 250 mm represents a threshold at which CBF is completely impaired (37).”

The corrected sentence appears below:

“It has been proposed that a diameter of 250 μm represents a threshold at which CBF is completely impaired (37).”

8. A correction should be made to **3 Pathophysiology: the immunothrombotic and endothelial responses to air emboli**, paragraph 3. The sentence previously stated:

“A typical result of air embolisms is the migration of air into small arteries with an average diameter range of 30–60 mm (2, 52).”

The corrected sentence appears below:

“A typical result of air embolisms is the migration of air into small arteries with an average diameter range of 30–60 μm.”

9. A correction has been made to **3.1 Thromboinflammation:hydrodynamics of bubble obstruction**, paragraph 1. The sentence previously stated:

“Yet for vessels with a radius greater than 100 mm, the bubble may continue to progress since the Laplace pressure of the bubble is less than 1 kPa which is insufficient to resist the driving pressure of the bubble (10–15 kPa in small arteries) (29, 120).”

The corrected sentence appears below:

“Yet for vessels with a radius greater than 100 μm, the bubble may continue to progress since the Laplace pressure of the bubble is less than 1 kPa which is insufficient to resist the driving pressure of the bubble (10–15 kPa in small arteries) (29, 120).”

10. A correction should be made to **3.2 Thromboinflammation: interaction of the endothelial glycocalyx with bubbles, red blood cells, platelets, activated neutrophils, coagulation and complement activation products, and subsequently, the release of cytokines**, paragraph 10. The sentence previously stated:

“Due to the varying sizes and deformity of the bubble along with temporal dispersion caused by HBOT and transmission of the bubble through arterioles less than 250 mm in size, there are varying levels of obstruction to blood flow that cause varying perturbations of the glycocalyx layer, which acts a natural surfactant (29, 165).”

The corrected sentence appears below:

“Due to the varying sizes and deformity of the bubble along with temporal dispersion caused by HBOT and transmission of the bubble through arterioles less than 250 μm in size, there are varying levels of obstruction to blood flow that cause varying perturbations of the glycocalyx layer, which acts a natural surfactant (29, 165).”

The authors apologize for these errors and state that these do not change the scientific conclusions of the article in any way. The original article has been updated.

